# A framework for simulating genotype-by-environment interaction using multiplicative models

**DOI:** 10.1007/s00122-024-04644-7

**Published:** 2024-08-06

**Authors:** J. Bančič, G. Gorjanc, D. J. Tolhurst

**Affiliations:** The Roslin Institute and Royal (Dick) School of Veterinary Studies, The University of Edinburgh, Easter Bush, Midlothian, UK

## Abstract

**Key message:**

**The simulation of genotype-by-environment interaction using multiplicative models provides a general and scalable framework to generate realistic multi-environment datasets and model plant breeding programmes.**

****Abstract**:**

Plant breeding has been historically shaped by genotype-by-environment interaction (GEI). Despite its importance, however, many current simulations do not adequately capture the complexity of GEI inherent to plant breeding. The framework developed in this paper simulates GEI with desirable structure using multiplicative models. The framework can be used to simulate a hypothetical target population of environments (TPE), from which many different multi-environment trial (MET) datasets can be sampled. Measures of variance explained and expected accuracy are developed to tune the simulation of non-crossover and crossover GEI and quantify the MET-TPE alignment. The framework has been implemented within the R package FieldSimR, and is demonstrated here using two working examples supported by R code. The first example embeds the framework into a linear mixed model to generate MET datasets with low, moderate and high GEI, which are used to compare several popular statistical models applied to plant breeding. The prediction accuracy generally increases as the level of GEI decreases or the number of environments sampled in the MET increases. The second example integrates the framework into a breeding programme simulation to compare genomic and phenotypic selection strategies over time. Genomic selection outperforms phenotypic selection by $\sim$50–70% in the TPE, depending on the level of GEI. These examples demonstrate how the new framework can be used to generate realistic MET datasets and model plant breeding programmes that better reflect the complexity of real-world settings, making it a valuable tool for optimising a wide range of breeding methodologies.

**Supplementary Information:**

The online version contains supplementary material available at 10.1007/s00122-024-04644-7.

## Introduction

Plant breeding is complicated by the fact that genotypes respond differently to different environments, a phenomenon known as genotype-by-environment interaction (GEI). Despite its importance, however, many current plant breeding simulations do not adequately capture the complexity of GEI because they either over-simplify it or ignore it completely. The framework developed in this paper simulates GEI with desirable structure using multiplicative models. The framework can be used to generate realistic multi-environment trial (MET) datasets and model plant breeding programmes that better reflect the complexity of real-world settings.

**Fig. 1:**
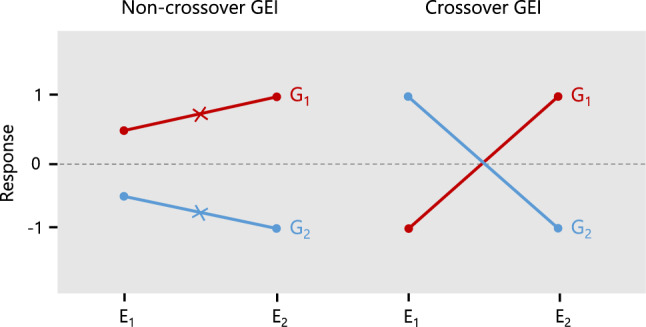
Response of genotypes G1 and G2 in environments E1 and E2 for a hypothetical continuous trait. The figure demonstrates genotype response in terms of non-crossover and crossover GEI, which reflect changes in scale and rank between environments. The *crosses* represent the genotype main effects (averages across environments)

Plant breeding has been historically shaped by GEI, from the selection of parents for future crosses to the development of commercial genotypes for release to growers. GEI can be broadly categorised as either non-crossover or crossover interaction, which reflect changes in the scale of genotype response between environments or changes in genotype rank (Fig. 1, Gail and Simon 1985; Baker 1988). Crossover GEI is of particular importance to breeders because their selection decisions are more complicated by changes in rank than changes in scale (Baker 1990; Eisemann et al. 1990). Plant breeders gauge the magnitude and form of GEI in their programmes by accumulating MET datasets, which contain a sample of environments that generally span multiple years and locations (Bernardo 2020). An important consideration when constructing a MET dataset is the extent to which it represents the breeder’s target population of environments (TPE, Comstock and Moll 1963; Cooper et al. 1993). This is referred to as the MET-TPE alignment (Cooper et al. 2023).

Multiplicative models have gained popularity in plant breeding because they are effective at capturing non-crossover and crossover GEI. The most general model for GEI is the *full rank* unstructured model, which fits a separate genetic variance for each environment and a separate genetic covariance for each pair of environments. The unstructured model captures the maximum amount of GEI in the data; however, it becomes computationally prohibitive and unnecessarily complicated as the number of environments increases. These issues can be overcome using *reduced rank* multiplicative models. The appealing feature of multiplicative models is that they generally capture a large proportion of GEI with a small number of multiplicative terms, where each term is the product of an environmental effect and a genotypic effect (Mandel 1971). Some traditional examples include AMMI (Kempton 1984; Gauch 1988), GGE (Cornelius et al. 1996; Yan et al. 2000) and factor analytic models (Piepho 1997; Smith et al. 2001). These approaches have been shown to provide an informative model for GEI and a good fit to MET datasets in general (Gauch et al. 2008; Kelly et al. 2007). The extensive theory and advantages of multiplicative models provide a good foundation for not only modelling GEI but also simulating it.

Simulations are routinely used in plant breeding as a fast and cost-effective tool for comparing different statistical approaches. Many studies have successfully generated MET datasets to address particular research objectives; however, they generally over-simplify GEI or are not suitable for scaling to a breeder’s TPE. For example, Hartung et al. (2023) simulated phenotypic data based on a simple variance component model for GEI, including components for genotype-by-year and genotype-by-location interaction (also see Krause et al. 2023; Arief et al. 2019). Variance component models do provide a scalable approach, but do not capture adequate genetic variance and covariance heterogeneity between environments (Piepho and Van Eeuwijk 2002; Smith et al. 2015). Another example is presented in Lisle (2023), who use factor analytic models to mimic the genetic variances and covariances observed in empirical data (also see Kelly et al. 2007; Nuvunga et al. 2019). They also consider relevant non-genetic effects, including spatial variation, but their approach is unnecessarily complicated and not well suited to scaling. The examples above provide motivation for a general framework for simulating GEI that is scalable to a breeder’s TPE, from which different MET datasets can be sampled.

Simulations are also routinely used to compare different breeding strategies over time (Bančič et al. 2023). Numerous simulation packages have been developed to model plant breeding programmes, including AlphaSimR (Gaynor et al. 2021), ADAM-Plant (Liu et al. 2019), ChromaX (Younis et al. 2023), GPOPSIM2 (Li et al. 2021), MOBPS (Pook et al. 2020) and QU-GENE (Podlich and Cooper 1998). These packages have been widely and successfully adopted for comparing different breeding strategies; however, most over-simplify GEI which can result in optimistic projections of genetic gain and spurious comparisons between breeding strategies. For example, AlphaSimR, ChromaX and GPOPSIM2 construct a single phenotype for each genotype comprising a genotype main effect, genotype-by-environment interaction effect and random error. The interaction effect is often modelled through a single multiplicative term, where the environmental effect is randomly sampled and consequently difficult to control (also see Gaynor et al. 2017; Bakare et al. 2024). However, most of these packages do have the functionality to simulate multiple environments as multiple correlated traits, so they do have the potential to implement a more realistic framework for GEI. This provides motivation for a general and scalable framework for simulating GEI within breeding programme simulations.

The aim of this paper is to develop a general framework for simulating GEI using multiplicative models. The framework can be used to simulate a set of environments that represent a breeder’s TPE, from which many different MET datasets can be sampled. Measures of variance explained and expected accuracy are developed to help tune the simulation of non-crossover and crossover GEI and quantify the MET-TPE alignment. The framework has been implemented within the R package FieldSimR (Werner et al. 2024), and is demonstrated here using two working examples supported by R code. The first example generates MET datasets with low, moderate and high GEI, which are then used to compare several popular statistical models. The second example integrates the framework into a breeding programme simulation to compare different selection strategies subject to different levels of GEI over time. Lastly, the framework has applications to simulating multiple TPE and multiple phenotypic traits as well as different biological genetic effects, including additive, dominance and epistasis.

## Material and methods

This section develops the framework for simulating GEI using multiplicative models. The framework is initially developed for simulating the genetic effects and then embedded within a linear mixed model to generate phenotypes which capture appropriate non-genetic effects. The framework has been implemented within the R package FieldSimR (Werner et al. 2024).

The methods consist of two parts:

1. Simulating genetic effects based on a between-environment genetic variance matrix. This matrix can be simulated with desirable structure representative of a hypothetical breeder’s TPE and tuned with measures of variance explained.
2. Generating phenotypes by combining the simulated genetic effects with non-genetic effects. Any number of MET datasets can be sampled from the TPE and summarised with measures of accuracy and MET-TPE alignment.

Each part is detailed in the following.

### Simulating genotype-by-environment effects

The framework is built on multiplicative models, which provide a general and scalable approach for simulating a wide range of GEI patterns. Assume the genetic effects are simulated for *v* genotypes in *p* environments, hereafter referred to as the genotype-by-environment (GE) effects. Let the *vp*-vector of GE effects be given by $\textbf{u}=({\textbf{u}}_1^{\!\scriptscriptstyle \top }, \ldots , {\textbf{u}}_p^{\!\scriptscriptstyle \top })^{\!\scriptscriptstyle \top }$, where ${\textbf{u}}_{j}$ is the *v*-vector for the $j^{th}$ environment. The GE effects are simulated as:

1 $$\begin{aligned} \textbf{u} \sim \text {N}\big (\textbf{0}, \mathbf {G_e} \otimes \textbf{G}\big ), \end{aligned}$$

where $\mathbf {G_e}$ is a $p \times p$ between-environment genetic variance matrix and $\textbf{G}$ is a $v \times v$ genotype relationship matrix. The matrix $\mathbf {G_e}$ may be estimated from empirical data or simulated, as demonstrated in the following section. The matrix $\textbf{G}$ is completely general and may represent a known/simulated pedigree or genomic relationship matrix generated through a breeding simulation package (see “Breeding programme simulation”). Both matrices are assumed to be positive (semi)-definite.

The GE effects are initially formulated according to an unstructured model and then reformulated according to a *reduced rank* multiplicative model. The unstructured model provides the most general form for simulating $\mathbf {G_e}$ based on a different genetic variance for each environment, $\sigma _{g_j}^2$, and a different genetic covariance for each pair of environments, $\sigma _{g_{ij}}$. The unstructured model can be written as a *full rank* multiplicative model with all *p* terms:

2 $$\begin{aligned} \textbf{u}&= \big (\textbf{s}_1 \otimes \textbf{f}_1\big ) + \ldots + \big (\textbf{s}_p \otimes \textbf{f}_p\big ) \nonumber \\&= \big (\textbf{S} \otimes \textbf{I}_v\big )\textbf{f}, \end{aligned}$$

where $\textbf{S} = [\textbf{s}_1 \, \ldots \, \textbf{s}_p]$ is a $p\times p$ matrix of environmental effects (covariates) and $\textbf{f}=({\textbf{f}}_1^{{\!\scriptscriptstyle \top }}, \ldots ,{\textbf{f}}_p^{{\!\scriptscriptstyle \top }} {)}^{{\!\scriptscriptstyle \top }}$ is a *vp*-vector of genotypic effects (slopes). The covariates and slopes are obtained from the eigendecomposition given by:

3 $$\begin{aligned} \textbf{G}_\textbf{e} = \textbf{U}\textbf{L}{\textbf{U}}^{{\!\scriptscriptstyle \top }}, \end{aligned}$$

where $\textbf{U}= [\textbf{u}_1 \, \ldots \, \textbf{u}_p]$ is an orthogonal matrix of eigenvectors and $\textbf{L}=\oplus _{r=1}^p l_r$ is a diagonal matrix of eigenvalues sorted in decreasing order. The covariates are set as $\textbf{S}=\textbf{U}$ and the slopes are simulated as $\textbf{f} \sim \text {N}(\textbf{0}, \, \textbf{L} \otimes \textbf{G})$. The proportion of genetic variance explained by the $r^{th}$ term can be calculated as $l_r/\sum ^p_{r=1} l_r$, where the denominator is equivalent to the sum of the diagonal elements of $\textbf{G}_\textbf{e}$ given by $\sum _{j=1}^p\sigma _{g_j}^2$. A large proportion of variance in MET data is typically explained by the first few terms, which makes the full rank form in Eq. (2) unnecessary as *p* increases.

The reduced rank form of Eq. (2) arises from taking the first *k* terms in Eq. (3), which gives:

4 $$\begin{aligned} \textbf{u} = \big (\textbf{S}_k \otimes \textbf{I}_v\big )\textbf{f}_k, \end{aligned}$$

where $\textbf{S}_{k}$ is a $p\times k$ matrix and $\textbf{f}_k$ is a *vk*-vector, with $\textbf{f}_k \sim \text {N}(\textbf{0}, \, \textbf{L}_k \otimes \textbf{G})$ such that ${\textbf {u}} \sim \text{ N }({\textbf {0}}, \, {\textbf {S}}_k{\textbf {L}}_k{{\textbf {S}}}_k^{\hspace{0.6pt}{\!\scriptscriptstyle \top }} \otimes {\textbf {G}})$ and ${\textbf {G}}_{\textbf {e}} = {\textbf {S}}_k{\textbf {L}}_k{{\textbf {S}}}_k^{\hspace{0.6pt}{\!\scriptscriptstyle \top }}$ has rank equal to *k*. The genotype slopes can be simulated independently or with a breeding simulation package by defining each multiplicative term as a separate trait with mean vector set to $\textbf{0}$, variance matrix set to $\textbf{L}_k$ and $\textbf{G}$ generated implicitly through the simulated population structure and genetic architecture of the trait/s (see “Breeding programme simulation”). The reduced rank model in Eq. (4) requires just *k* traits (terms) to be simulated, which makes it substantially more computationally efficient than the full rank model. It also provides motivation to pre-simulate a reduced rank form of $\textbf{G}_\textbf{e}$ with rank *k*, as demonstrated in the following section.

#### Simulating a between-environment genetic variance matrix

The between-environment genetic variance matrix is simulated with desirable structure that is both controllable and commensurate with the reduced rank model in Eq. (4). This is achieved by simulating heterogeneous genetic variances and correlations through:

5 $$\begin{aligned} \mathbf {G_e} = \textbf{D}_{\textbf{e}}^{1/2}\mathbf {C_e}\textbf{D}_{\textbf{e}}^{1/2}, \end{aligned}$$

where $\mathbf {D_e}$ is a $p \times p$ diagonal genetic variance matrix with diagonal elements given by $\sigma _{g_j}^2$ and $\mathbf {C_e}$ is a $p \times p$ reduced rank between-environment genetic correlation matrix with off-diagonal elements given by $\rho _{g_{ij}} = \sigma _{g_{ij}}/\sigma _{g_i}\sigma _{g_j}$.

**Fig. 2:**
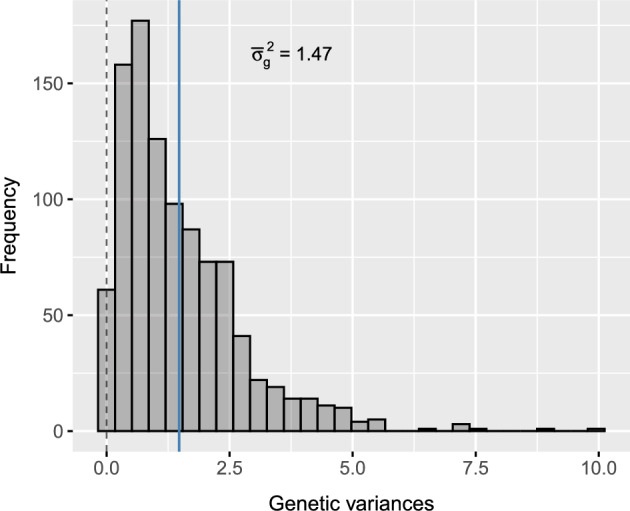
Simulated genetic variances for 1000 environments, obtained by sampling from a gamma distribution with shape of 1.5 and scale of 1. The *solid line* represents the mean genetic variance (also labelled). Note: The heterogeneity of genetic variance is $\sigma ^2_{ge_h} = 0.23$

The genetic variances in $\mathbf {D_e}$ are simulated as $\sigma ^2_{g_j} \sim \text {Gamma}(\alpha ,\theta )$, where $\alpha$ is the shape parameter and $\theta$ is the scale parameter. Note that $\alpha \theta$ approaches the mean genetic variance across environments given by $\bar{\sigma }_g^2=\sum _{j=1}^p\sigma _{g_j}^2/p$ when *p* is sufficiently large. These parameters are set to $\alpha =1.5$ and $\theta =1$ for all examples in this paper (Fig. 2), but note that other values can be used where required. The gamma distribution was chosen here because it produces positive genetic variances with a skewed distribution and realistic heterogeneity.

Applying Hardin et al. (2013), the reduced rank between-environment genetic correlation matrix is simulated as:

6 $$\begin{aligned} \mathbf {C_e} = \rho \textbf{J}_p + \epsilon \varvec{\varLambda }^{{\!\scriptscriptstyle \top }} \varvec{\varLambda }, \end{aligned}$$

where $\rho$ is the baseline genetic correlation, $\textbf{J}_p$ is a $p\times p$ matrix of ones, $\epsilon$ controls the variability of the correlations (magnitude of structured noise) around the baseline and $\varvec{\varLambda } = [\varvec{\varLambda }_1 \, \ldots \, \varvec{\varLambda }_p]$ is a $(k-1)\times p$ matrix of latent covariates in which $\varvec{\varLambda }_{j}$ is the vector for the $j^{th}$ environment and $k\ge 2$. The reduced rank form of $\mathbf {C_e}$ arises from the fact that $\textbf{J}_p$ has rank 1 and $\varvec{\varLambda }$ has rank $k-1$, or more specifically that $\rho \textbf{J}_p + \epsilon \varvec{\varLambda }^{{\!\scriptscriptstyle \top }} \varvec{\varLambda }$ has rank *k* when $\rho , \epsilon \ne 0$. Note that other base correlation functions can be used instead of $\rho {\textbf {J}}_p$, including autoregressive processes, variance component models capturing genotype-by-year and genotype-by-location interaction and block structures capturing multiple phenotypic traits or multiple TPE (see “Appendix A”).

The baseline correlation is subject to the constraint $0 \le \rho < 1$, which ensures $\mathbf {C_e}$ is positive (semi)-definite. If the constraint is not imposed and $-1< \rho < 0$, indefinite matrices may be generated that require bending. The noise is also subject to the constraint $\epsilon = 1 - \rho$, which ensures that the rank of $\mathbf {C_e}$ equals *k* when $0< \rho < 1$. If the constraint is not imposed and $\epsilon < 1 - \rho$, the diagonal must be constrained to one, and the rank of $\mathbf {C_e}$ will equal *p*. The first *k* terms will still capture the majority of variation in $\mathbf {G_e}$, but now the remaining $p-k$ terms will each capture a small proportion of variance given by $1 - \rho - \epsilon$.

An extension of Hardin et al. (2013) is used to simulate the genetic correlations, with new functionality to control the skewness of their distribution. The columns of $\varvec{\varLambda }$ in Eq. (6) are simulated as $\varvec{\varLambda }_{j} \sim \text {U}(-1, 1 + \gamma ),$ where $\gamma$ governs the amount of negative skewness and $-1 \le \gamma \le 0$. The $\varvec{\varLambda }_{j}$ are then scaled to unit length, i.e. $\varvec{\varLambda }_{j}^{{\!\scriptscriptstyle \top }}\varvec{\varLambda }_{j} = 1$. Note that when $\gamma =0$, the baseline correlation $\rho$ approaches the mean genetic correlation between environments given by $\bar{\rho }_g= 2\sum _{i < j}^p \rho _{g_{ij}}/p(p-1)$, but not when $\gamma \ne 0$.

Different structure in $\mathbf {C_e}$ can be generated by altering $\rho$, $\epsilon$, $\gamma$ and *k*. The examples in Supplementary Fig. 1 demonstrate that decreasing $\rho$ decreases the mean genetic correlation between environments, increasing $\epsilon$ increases the variability of the correlations around the mean, altering $\gamma$ changes the negative skewness of the distribution, and increasing *k* increases the rank of the noise and thence decreases the amount of structure.

**Fig. 3:**
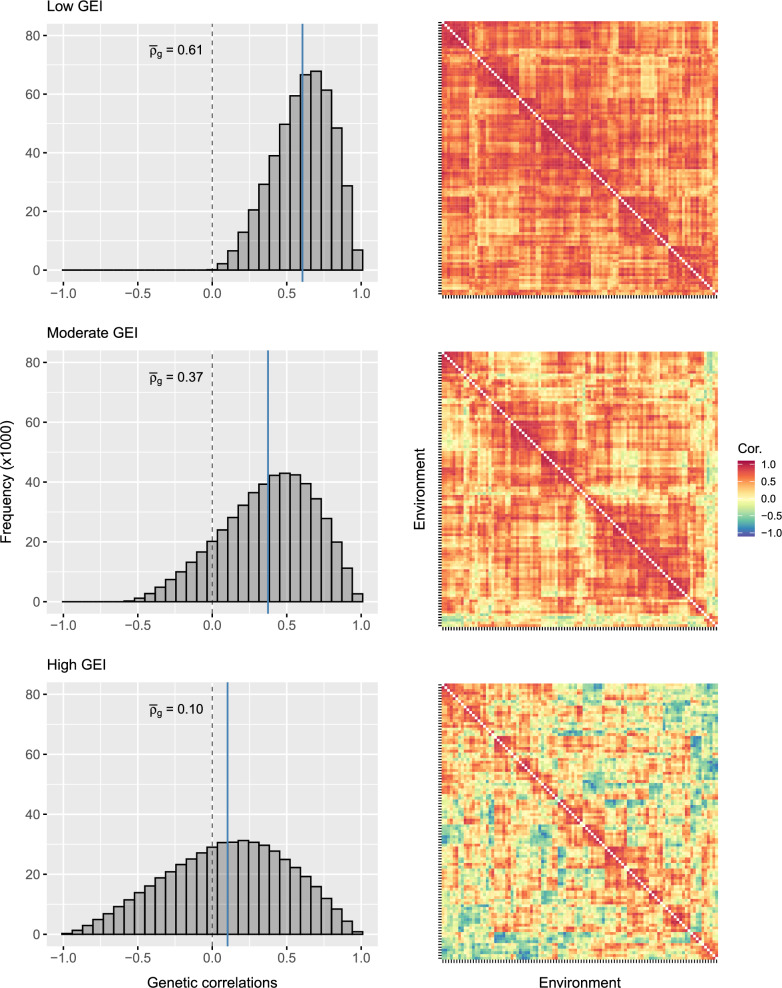
Simulated between-environment genetic correlation matrices with low, moderate and high GEI for 1000 environments. The *solid lines* on the histograms represent the mean genetic correlation (also labelled). The *colour key* for the heatmaps ranges from 1 (agreement in rankings) through 0 (disagreement in rankings) to $-1$ (reversal of rankings). Note: The heatmaps have been reduced to the first 100 environments for display. All matrices are hierarchically ordered based on separate dendrograms

The framework above was used to simulate the three examples of $\mathbf {C_e}$ presented in Fig. 3 and summarised in Table 1. The matrices were constructed by tuning $\rho$, $\epsilon$ and $\gamma$ using the measures of variance explained developed below. All matrices were simulated with rank $k=7$ because this imposed sufficient structure for demonstration, but *k* should be altered as required. All matrices were then multiplied with $\mathbf {D_e}$ in Fig. 2 to create three examples of $\mathbf {G_e}$ using Eq. (5). These matrices form the basis of the low, moderate and high GEI scenarios used throughout the remainder of the paper. Supplementary Script S1 provides functionality to simulate $\mathbf {G_e}$ with desirable structure using FieldSimR.

**Table 1:** Summary of the simulated between-environment genetic variance matrices with low, moderate and high GEI

GEI	Input parameters						Output parameters					
	$\alpha$	$\theta$	$\rho$	$\epsilon$	$\gamma$	*k*	$v_g$	$v_{ge}$	$v_n$	$v_c$	$\bar{\sigma }^2_g$	$\bar{\rho }_{g}$
Low	1.5	1	0.50	0.50	$-$0.50	7	0.51	0.49	0.61	0.39	1.47	0.61
Moderate	1.5	1	0.20	0.80	$-$0.50	7	0.32	0.68	0.37	0.63	1.47	0.37
High	1.5	1	0.00	1.00	$-$0.35	7	0.08	0.92	0.09	0.91	1.47	0.10

Presented are the shape ($\alpha$) and scale ($\theta$) parameters for simulating $\mathbf {D_e}$ in Fig. 2 and the baseline genetic correlation ($\rho$), magnitude ($\epsilon$) and skewness ($\gamma$) of noise and the rank (*k*) for simulating $\mathbf {C_e}$ in Fig. 3. Also presented are the proportions of main effect ($v_g$) and interaction ($v_{ge}$) variance and non-crossover ($v_n$) and crossover ($v_c$) variance simulated in $\mathbf {G_{e}}$ as well as the mean genetic variance ($\bar{\sigma }^2_g$) and correlation ($\bar{\rho }_g$)

#### Measures of variance explained

Measures of variance explained are developed in the following to quantify and tune the proportions of (i) main effect and interaction variance and (ii) non-crossover and crossover variance simulated in $\mathbf {G_e}$. Further derivations of the measures are provided in “Appendix B”, including the calculation of genotype main effects from the multiplicative model in Eq. (4) as averages across environments.

1. The proportion of main effect variance is:

7 $$\begin{aligned} v_g = \frac{\sigma ^2_g}{\sigma ^2_g + \sigma ^2_{ge}}, \end{aligned}$$

where $\sigma ^2_g$ is the main effect variance and $\sigma ^2_{ge}$ is the interaction variance, which are equal to the mean element of $\mathbf {G_e}$ and the mean diagonal element of $\mathbf {G_e}-\sigma ^2_g\textbf{J}_p$, respectively. The proportion of interaction variance is, therefore, given by $v_{ge} = 1 - v_g$. The interaction variance can be further partitioned into heterogeneity of genetic variance, $\sigma ^2_{ge_h}$, and lack of genetic correlation, $\sigma ^2_{ge_l}$ (see “Appendix B”).

2. The proportion of non-crossover variance is:

8 $$\begin{aligned} v_n = \frac{\sigma ^2_n}{\sigma ^2_g + \sigma ^2_{ge}}, \end{aligned}$$

where $\sigma ^2_{n}$ is the non-crossover variance, which quantifies the genetic variation in each environment attributed to perfect positive correlation with the genotype main effects, i.e. $\sigma ^2_n= \sum _{j=1}^p \rho ^{*2}_{g_j}\sigma ^2_{g_j}/p$, where $\rho ^*_{g_j}$ is the correlation between the main effects and GE effects for the $j^\textrm{th}$ environment. The crossover variance then quantifies all remaining variation in the GE effects which arises from a lack of perfect positive correlation with the genotype main effects (Fig. 1, also see Tolhurst 2024). The proportion of crossover variance is, therefore, given by $v_{c} = 1 - v_n$.

The measures of variance explained are demonstrated for the three examples of $\mathbf {G_e}$ summarised in Table 1. The examples were classified as the low, moderate and high GEI scenarios by tuning the proportions of non-crossover and crossover variance. It is important to note that the examples targeted non-crossover proportions of $\sim 0.6$, 0.4 and 0.1 for the three scenarios, which captured $\sim 20\%$ more variation relative to the main effect variance (with $\sigma ^2_n-\sigma ^2_g\approx 0.10$, 0.05 and 0.01, respectively). This was achieved by tuning the baseline correlation and skew in $\mathbf {C_e}$, as well as the shape and scale parameters of the gamma distribution. All examples have the same heterogeneity of genetic variance, ${\sigma }_{ge_h}^2 = 0.23$, but differing lack of genetic correlation given by ${\sigma }_{ge_l}^2 = 0.49, 0.77$ and 1.12, respectively. Supplementary Script S1 provides functionality to obtain the measures of variance explained using FieldSimR.

### Simulating phenotypes

**Fig. 4:**
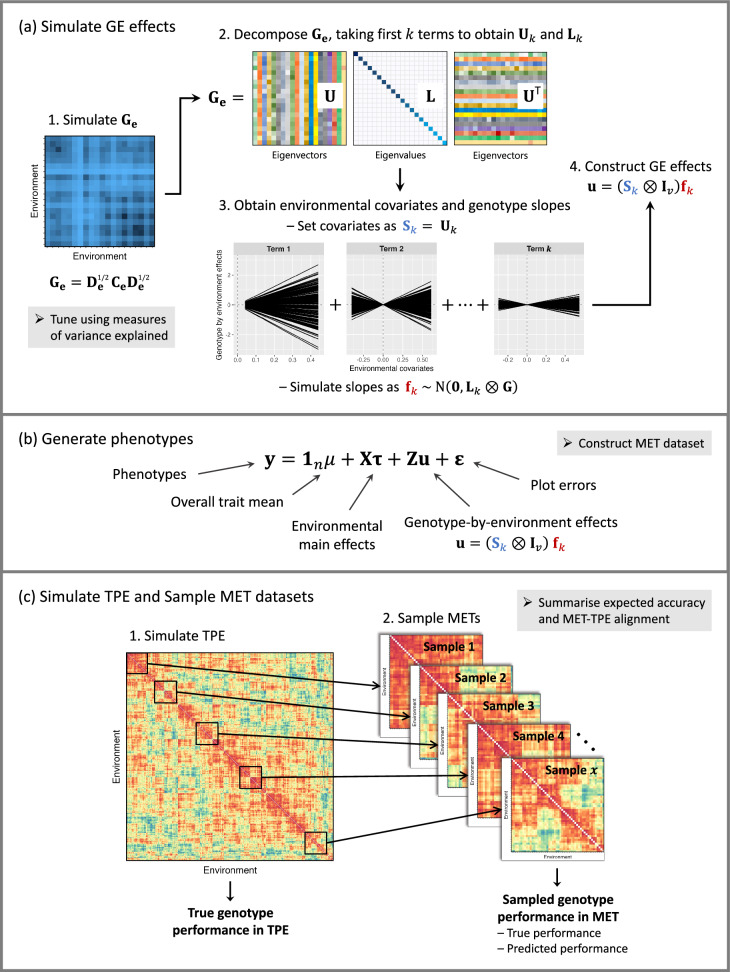
Overview of the framework for simulating GEI using multiplicative models. Presented are **a** the four key steps for simulating the genotype-by-environment (GE) effects, **b** how the GE effects are embedded within a linear mixed model to generate phenotypes and **c** the process of sampling MET datasets from a simulated TPE, represented by a between-environment genetic correlation matrix. Note: The TPE is shuffled prior to sampling each MET dataset in order to capture the full extent of GEI

The framework for simulating the GE effects can be embedded within a linear mixed model to generate phenotypes which also capture appropriate non-genetic effects. The framework is summarised in Fig. 4, with R code provided in “Appendix C” for a small working example and in Supplementary Script S2 for generating a realistic MET dataset with FieldSimR.

Let the *n*-vector of phenotypes be given by $\textbf{y}=({\textbf{y}}_1^{\!\scriptscriptstyle \top }, \ldots , {\textbf{y}}_p^{\!\scriptscriptstyle \top })^{\!\scriptscriptstyle \top }$, where ${\textbf{y}}_{j}$ is the $n_j$-vector for the $j^{th}$ environment. The linear mixed model used to simulate $\textbf{y}$ is given by:

9 $$\begin{aligned} \textbf{y} = \textbf{1}_n\mu + \textbf{X}\varvec{\tau } + \textbf{Z}\textbf{u} + \varvec{\varepsilon }, \end{aligned}$$

where $\mu$ is the overall trait mean, $\varvec{\tau }$ is a *p*-vector of environmental main effects with $n \times p$ design matrix $\textbf{X}$, $\textbf{u}$ is the *vp*-vector of GE effects with $n \times vp$ design matrix $\textbf{Z}$ and $\varvec{\varepsilon }$ is the *n*-vector of plot errors. A randomised complete block design is used in the example R code with *r* replicate blocks, but note that simulation of other experimental designs and additional non-genetic effects is encouraged.

The environmental main effects are simulated as:

10 $$\begin{aligned} \varvec{\tau } \sim \text {N}(\textbf{0}, \sigma _{e}^2\textbf{I}_p), \end{aligned}$$

where $\sigma _{e}^2$ is the environmental main effect variance. The environmental main effects can also be extended to a regression on environmental covariates as $\varvec{\tau }= \textbf{S}_k \varvec{\tau }_{\textbf{s}_k}$, where $\varvec{\tau }_{{\textbf {s}}_k}$ is a *k*-vector with elements given by the mean response of genotypes to each covariate. This extension induces a mean-variance ratio and enables genetic gain to be tracked in a breeding programme simulation (see “Breeding programme simulation”).

The GE effects are simulated using the framework in “Simulating genotype-by-environment effects”, which can be summarised by four key steps:

1. Simulate a between-environment genetic variance matrix as $\mathbf {G_e} = \textbf{D}_{\textbf{e}}^{1/2}\mathbf {C_e}\textbf{D}_{\textbf{e}}^{1/2}$, tuned using the measures of variance explained.
2. Decompose the between-environment genetic variance matrix as $\textbf{G}_\textbf{e} = \textbf{U}\textbf{L}{\textbf{U}}^{{\!\scriptscriptstyle \top }}$, taking the first *k* terms to obtain $\textbf{U}_k$ and $\textbf{L}_k$.
3. Set the environmental covariates as $\textbf{S}_k = \textbf{U}_k$ and simulate the genotype slopes as $\textbf{f}_k \sim \text {N}(\textbf{0}, \, \textbf{L}_k \otimes \textbf{G})$, either independently or with a simulation package.
4. Construct the GE effects as $\textbf{u} = \big (\textbf{S}_k \otimes \textbf{I}_v)\textbf{f}_k$.

The framework can be used to generate additive, dominance and epistatic GE effects by appropriately defining $\mathbf {G_e}$ and $\textbf{G}$, either explicitly or through simulated population structure and trait architecture.

Lastly, the plot errors are simulated as:

11 $$\begin{aligned} \varvec{\varepsilon } \sim \text {N}(\textbf{0}, \sigma ^2_{\varepsilon }\textbf{R}), \end{aligned}$$

where $\sigma ^2_{\varepsilon }$ is the mean error variance across environments and $\textbf{R}$ is the error variance matrix, which is asumed to be completely general. Supplementary Script S2 demonstrates FieldSimR’s functionality to generate correlated plot errors which capture spatial variation.

#### Simulating a breeder’s TPE and sampling a MET dataset

The framework can be used to simulate a set of environments that represent a hypothetical breeder’s TPE, from which many different MET datasets can be sampled. This process is summarised in Fig. 4c for the between-environment genetic correlation matrix, $\mathbf {C_e}$, and demonstrated in “Appendix C” using base R functions and Supplementary Scripts S1 and S2 using FieldSimR. Assume the vector of GE effects, $\textbf{u}$, includes all *p* environments in the hypothetical TPE, where $p=1000$ for demonstration, but this can be altered as required. A subset of $p_m$ environments is sampled from the TPE to represent the natural sampling which occurs during field evaluation, with GE effects denoted by $\textbf{u}_m$. Phenotypes are then constructed for the environments sampled in the MET dataset by adding appropriate non-genetic effects to $\textbf{u}_m$ following Eq. (9). The MET-TPE alignment for each sample is quantified using the empirical correlation between the genotype main effects in the MET and the TPE (Cooper et al. 2023). Sampling MET datasets in this manner will be demonstrated for the breeding programme simulation in “Breeding programme simulation”, but note that the sampling process is completely general and can be tailored to many different plant breeding scenarios.

#### Measures of expected accuracy

Measures of accuracy are developed in the following to quantify the expected correlation between the (i) predicted genotype main effects obtained from the MET dataset and the true genotype main effects in the TPE and MET, referred to as the expected main effect accuracy, (ii) true genotype main effects in the MET dataset and TPE, i.e. the expected MET-TPE alignment, and (iii) predicted and true GE effects in the MET dataset. The measures below are the expected (positive) correlations based on the true simulation parameters.

1. The expected main effect accuracy in the TPE is:

12 $$\begin{aligned} r_g = \sqrt{\frac{\sigma ^2_g}{\sigma ^2_g + \sigma ^2_{ge}/p_m + {\sigma }^2_{\varepsilon }/p_mr}}, \end{aligned}$$

which is equal to the square root of the line-mean heritability across environments (Cooper and DeLacy 1994).

The expected main effect accuracy in the MET dataset is:

13 $$\begin{aligned} r_m = \sqrt{\frac{\sigma ^2_g + \sigma ^2_{ge}/p_m}{\sigma ^2_g + \sigma ^2_{ge}/p_m + {\sigma }^2_{\varepsilon }/p_mr}}, \end{aligned}$$

where $\sigma ^2_g + \sigma ^2_{ge}/p_m$ is the expected genotype main effect variance sampled in the MET dataset. This variance arises from an inflation of the true main effect variance in Eq. (12) by $\sigma ^2_{ge}/p_m$, which represents the sampling error in the MET dataset. The practical implication is that the expected accuracy for the sampled MET dataset will always be higher than for the full TPE, provided $\sigma ^2_{ge}\ne 0$, i.e. $r_m \ge r_g$.

2. The expected MET-TPE alignment is:

14 $$\begin{aligned} r_{mt} = \sqrt{\frac{\sigma ^2_g}{\sigma ^2_g + \sigma ^2_{ge}/p_m}}, \end{aligned}$$

which is obtained by setting ${\sigma }^2_{\varepsilon } = 0$ in Eq. (12). This measure will be used in a MET dataset simulation as an indicator to the maximum main effect accuracy in the TPE (see “Model comparison”).

The fundamental relationship between Eqs. (12), (13) and (14) is then given by (Cooper et al. 2023):

15 $$\begin{aligned} r_g = r_m \times r_{mt}, \end{aligned}$$

such that the expected main effect accuracy in the TPE is equal to the expected accuracy in the MET dataset multiplied by the MET-TPE alignment.

3. The expected accuracy of the GE effects in the MET dataset is:

16 $$\begin{aligned} r_{ge} = \sqrt{\frac{\sigma ^2_g + \sigma ^2_{ge}}{\sigma ^2_g + \sigma ^2_{ge} + {\sigma }^2_{\varepsilon }/r}}, \end{aligned}$$

which is equal to the square root of the line-mean heritability within environments.

The measures of accuracy are presented in Supplementary Fig. 2a–c for different values of $\sigma ^2_g$, $\sigma ^2_{ge}$ and $p_m$. These figures show that increasing the number of environments sampled in the MET dataset will increase the MET-TPE alignment and decrease the sampling error, to a point where the contribution of the interaction variance becomes negligible. The measures of accuracy will be used to inform the MET dataset and breeding programme simulations in “MET dataset simulation” and “Breeding programme simulation”.

### Implementation within FieldSimR

The framework for simulating GEI developed above has been implemented within the R package FieldSimR (Werner et al. 2024), which is available on CRAN. FieldSimR offers functionality to simulate, tune and visualise a reduced rank between-environment genetic variance matrix, $\mathbf {G_e}$, by generating user-defined structures for the genetic variances and correlations between environments. Wrapper functions are provided which decompose $\mathbf {G_e}$ to generate GE effects using AlphaSimR (Gaynor et al. 2021) with desired population structure and trait architecture for any number of multiplicative terms. FieldSimR also offers functionality to simulate and sample environments from a TPE, representing a MET, which can be summarised using measures of accuracy and MET-TPE alignment. Phenotypes are constructed by combining the simulated GE effects with plot errors that capture spatial variation. Example scripts demonstrating the functionality of FieldSimR are provided in the Supplementary Material.

## Results

The following sections showcase the application of the new framework by simulating realistic MET datasets and by integrating the framework into a breeding programme simulation. The MET datasets are used to compare several popular statistical models while the breeding programme simulation is used to compare different selection strategies, both with three different levels of GEI. Examples are also provided in the Supplementary Material which demonstrate the functionality of FieldSimR (Werner et al. 2024). It is important to note that the primary objective of this section is to highlight the utility of the new framework rather than to make specific recommendations.

**Table 2:** Summary of the example MET dataset simulated using the base R code in “Appendix C”

Env	Design			Trait			
	Genos	Blocks	Plots	Mean	$\sigma _{g_j}^2$	$\sigma _{\varepsilon _j}^2$	$H_j^2$
E1	400	2	800	3.61	4.81	3.94	0.55
E2	400	2	800	3.52	4.00	4.13	0.49
E3	400	2	800	4.16	0.34	4.01	0.08
E4	400	2	800	2.50	0.39	4.05	0.09
E5	400	2	800	4.83	5.86	4.03	0.59
E6	400	2	800	5.54	1.08	3.81	0.22
E7	400	2	800	3.35	0.07	3.96	0.02
E8	400	2	800	4.13	3.01	4.34	0.41
E9	400	2	800	5.36	1.60	3.74	0.30
E10	400	2	800	3.00	2.63	4.28	0.38
**Overall**	**400**	**2**	**8000**	**4**.**00**	**2**.**38**	**4**.**03**	**0**.**31**

Presented are the number of genotypes, blocks and plots in each environment. Also presented for a hypothetical continuous trait are the environment means ($\mu + \tau _j$), genetic variances ($\sigma _{g_j}^2$), error variances ($\sigma _{\varepsilon _j}^2$) and plot-level heritabilities ($H_j^2$). The MET-TPE alignment for this sample of environments is $r_{mt}=0.93$

### MET dataset simulation

This section simulates a small example using the framework developed in “Simulating genotype-by-environment effects”, which can be reproduced with the base R code in “Appendix C”. GE effects are generated for $v=400$ genotypes in a small hypothetical TPE with $p=100$ environments. The GE effects are simulated with genetic variances sampled from a gamma distribution and genetic correlations sampled from a continuous uniform distribution. A MET dataset is sampled from the TPE with $p_m=10$ environments, producing a MET-TPE alignment of $r_{mt}=0.93$ (Table 2). A randomised complete block design is used for each environment with $r=2$ replicate blocks of 400 plots. Phenotypes are then constructed by adding environmental main effects and plot errors for a hypothetical continuous trait with mean of 4. The environmental main effects are sampled from a standard normal distribution and the plot errors are simulated assuming independence and an overall plot-level heritability of 0.3. This produces heterogeneous environment means, variances and heritabilities.

The small example was extended using FieldSimR functionality. Supplementary Script S1 demonstrates the simulation of a reduced rank between-environment genetic variance matrix representative of a hypothetical TPE, now with $p = 1000$ environments, which is tuned using the measures of variance explained from “Measures of variance explained”. Supplementary Script S2 then demonstrates the simulation of a MET dataset by sampling $p_m=20$ environments from the TPE. AlphaSimR is used to simulate additive GE effects with desired population structure and trait architecture for seven multiplicative terms. FieldSimR is used to generate phenotypes by combining the simulated GE effects with correlated plot errors based on a randomised complete block design. Lastly, the measures of accuracy and MET-TPE alignment from “Measures of expected accuracy” are used to summarise the simulated MET dataset.

#### Model comparison

This section demonstrates how different statistical models can be compared using simulated MET datasets built on the examples above. Three hypothetical TPEs were simulated with low, moderate and high GEI, each with $p=1000$ environments in total. The scenarios correspond to the between-environment genetic variance matrices presented in Figs. 2 and  3 and summarised in Table 1. Four MET datasets were constructed for each level of GEI by randomly sampling $p_m=5, 10, 20$ and 50 environments from each TPE (Fig. 4). This process was replicated 1000 times, with eight statistical models fitted to each replicate. The eight models included main effects, compound symmetry, main effects plus diagonal, diagonal and factor analytic of order 1–4. Further details are provided in the Supplementary Material (also see Tolhurst et al. 2022). The aim of the analyses was to predict the genotype main effects and GE effects within each environment. All models were fitted using ASReml-R (Butler et al. 2017), as demonstrated in Supplementary Script S3.

The true parameters for all 1000 replicates are summarised in Supplementary Fig. 3a–c. These figures compare the true parameters in each TPE with those sampled in the MET datasets. The parameters become more aligned to the TPE as more environments are sampled. This is the case for all levels of GEI, but note that fewer environments are required for the simulated parameters to become well aligned for the low GEI scenario compared to the moderate and high GEI scenarios.

**Fig. 5:**
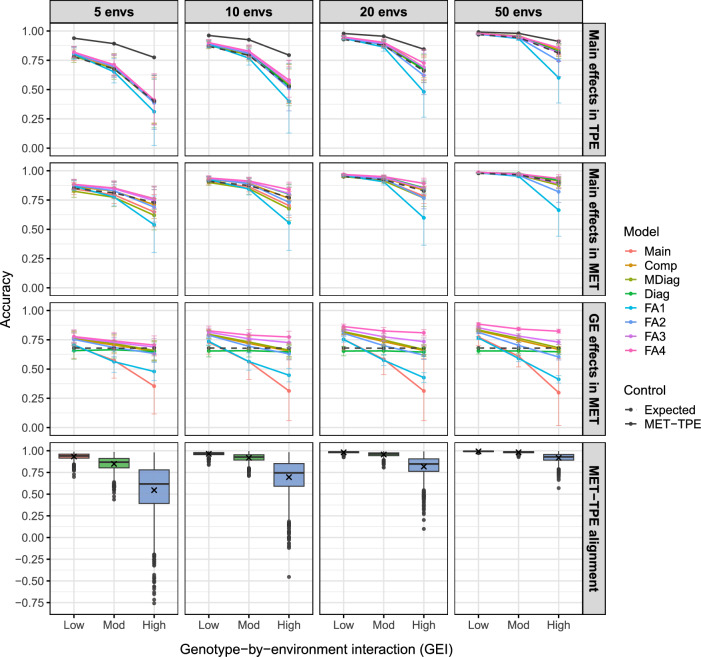
Eight statistical models fitted to simulated MET datasets with 5, 10, 20 or 50 environments and low, moderate or high GEI. The top two panels show the main effect accuracy in the TPE and MET dataset while the third panel shows the accuracy of the predicted GE effects in the MET, with error bars representing 80% sample quantiles. The bottom panel shows the MET-TPE alignments for all 1000 simulation replicates, with *crosses* representing the expected alignments. Note: The genotype main effects in all factor analytic models were obtained as averages across environments. The factor analytic models of order three and four were fitted without the diagonal term for the 5 environment scenarios. Main—main effects, Comp—compound symmetry, MDiag—main effects plus diagonal, Diag—diagonal, FA—factor analytic

Figure 5 presents the prediction accuracy of the eight statistical models fitted to simulated MET datasets with different levels of GEI and different numbers of environments. This figure also includes the expected main effect accuracy in the MET dataset and TPE (*dashed black lines*), and the expected MET-TPE alignment (*solid black line*) from “Measures of expected accuracy”. There are five general results:

1. All prediction accuracies decrease as the level of GEI increases. The largest differences occur between models at high GEI.
2. All prediction accuracies increase as the number of sampled environments increases. The largest differences occur between models at five environments for the genotype main effects and at 50 environments for the GE effects, particularly for high GEI.
3. The main effect accuracies are higher in the MET than in the TPE. The smallest differences occur between models at 50 environments, where the sampled MET datasets become more aligned to the TPE.
4. The main effect accuracies in the MET dataset are highest for the factor analytic models of order three and four, but the differences become negligible in terms of the TPE (except for the factor analytic model of order one).
5. The prediction accuracies for the GE effects in the MET dataset are also highest for the factor analytic models of order three and four. The largest differences occur between models at high GEI and 50 environments.

The MET-TPE alignments for all 1000 simulation replicates are also presented in Fig. 5. This figure provides further insight into the extent and form of the GEI simulated in the TPE and sampled in the MET datasets. Not only does the alignment decrease as the level of GEI increases or the number of sampled environments decreases, but the variability around the expected alignment also increases. For example, very well-aligned or very poorly aligned MET datasets can be constructed with as few as five environments, depending on the level of GEI. Optimising the construction of well-aligned MET datasets is the topic of active research.

Key summaries for the eight statistical models are presented in Supplementary Fig. 4a–b. These figures include measures of reliability and model fit for the 1000 simulation replicates. The factor analytic models generally produce the most reliable estimates of the genetic variances and covariances in terms of root-mean-square error. They also generally provide a superior fit to the data in terms of AIC and proportion of variance explained, but have substantially longer running times.

**Fig. 6:**
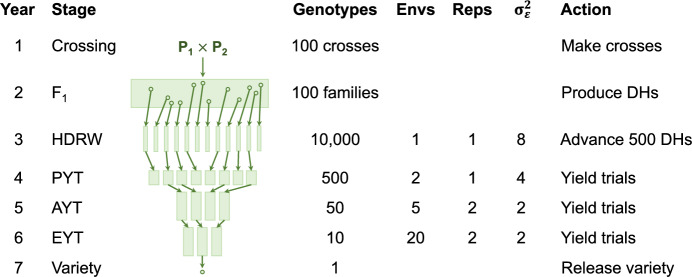
Key features of the simulated plant breeding programme. Presented are the number of genotypes, environments and replicates per environment as well as the mean error variance ($\sigma _\varepsilon ^2$) and the action taken. DH—double haploid, FS—full sib, HDRW—headrow, PYT—preliminary yield trial, AYT—advanced yield trial, EYT—elite yield trial

Although the results above are well aligned with previous empirical and simulation studies (see, for example, Crossa et al. 2006; Kelly et al. 2007; Burgueño et al. 2008; Smith et al. 2015; Bakare et al. 2022), it is important to reiterate that the primary objective here was to demonstrate the potential application of the new framework for comparing statistical approaches, rather than make specific recommendations for using factor analytic models or for using specific forms/orders of these models (Piepho 1997; Smith et al. 2001).

### Breeding programme simulation

This section integrates the framework developed in “Simulating genotype-by-environment effects” into a breeding programme simulation. The three hypothetical TPE from “MET dataset simulation” are again used for demonstration. The simulation involves 20 years of breeding for a hypothetical continuous trait, with 20 environments randomly sampled from the 1000 environments in each TPE every year (Fig. 4c). There are four stages of field evaluation, with an increasing number of environments and a decreasing number of genotypes observed in each stage (Fig. 6). This produces a subset of 400 environments from each TPE and a maximum of 20 environments observed in each stage, every year.

The following workflow was developed to integrate the framework into a simulation package:

1. *Simulate and decompose* a between-environment genetic variance matrix representing a hypothetical TPE. This produces the full set of environmental covariates in the TPE, denoted by $\textbf{S}_k$.
2. *Simulate genotype slopes* in a founder population, denoted by $\textbf{f}_k$. This is achieved by defining each multiplicative term as a separate trait in the simulation package. The traits (terms) are simulated with mean vector set to $\textbf{0}$ and variance matrix set to $\textbf{L}_k$.
3. *Sample a subset of environments* from the TPE each year of breeding. The sample of environments can be ordered and structured as required to represent specific trial sites.
4. *Construct GE effects and phenotypes*. The true GE effects in the TPE are constructed by multiplying $\textbf{S}_{k}$ from Step 1 with the current genotype slopes, denoted by $\textbf{f}^*_k$. Phenotypes are constructed for the environments sampled in the current year by adding error.
5. *Select and advance superior genotypes* based on predicted GE effects obtained from analysing a MET dataset. The MET dataset may contain any number of environments spanning any number of years, locations and stages.
6. *Track genetic progress* via genetic gain, genetic variance and measures of accuracy. These measures are based on the GE effects in the TPE and MET dataset, and are reflective of changes in the sampled environments and breeding population over time.

Steps 1 and 2 are performed once at the beginning of every simulation while Step 3 is performed once every year and Steps 4–6 are performed in each stage, every year for 20 years of breeding.

Genetic progress is tracked for each stage during Step 6. The genetic gain in the demonstration below is taken as the mean of the genotype main effects, which is given by $\mu _g^*=\bar{{\textbf {s}}}_k\varvec{\tau }^*_{{\textbf {s}}_k}$ for the TPE, where $\bar{{\textbf {s}}}_k$ is a *k* row-vector of means for each environmental covariate and $\varvec{\tau }^*_{{\textbf {s}}_k}$ contains the means of the current genotype slopes. The genetic variance is then taken as the variance of the genotype main effects, which is given by $\sigma ^{*2}_{g} = \bar{{\textbf {s}}}_k{\textbf {L}}^*_k\bar{{\textbf {s}}}_k^{\hspace{1.0pt}{\!\scriptscriptstyle \top }}$, where ${\textbf {L}}^*_k$ contains the variances and covariances of the current genotype slopes. Initially, $\mu _g=0$ and $\sigma ^2_{g} = \bar{{\textbf {s}}}_k{\textbf {L}}_k\bar{{\textbf {s}}}_k^{\hspace{1.0pt}{\!\scriptscriptstyle \top }}$ since ${\textbf {f}}_k \sim \text{ N }({\textbf {0}}, \, {\textbf {L}}_k \otimes {\textbf {G}})$ in the founder population, but note that an initial overall mean can be included where required. Similar measures are obtained for the MET dataset using the subset of environments sampled in each MET.

Example R code for integrating the framework into AlphaSimR (Gaynor et al. 2021) using FieldSimR (Werner et al. 2024) functionality is provided in Supplementary Scripts S4, but note that the workflow above can be integrated into many current simulation packages to efficiently simulate GEI at a very large scale. The examples in this paper highlight the efficiency of the new framework, with just seven traits simulated instead of the 400 otherwise required.

**Fig. 7:**
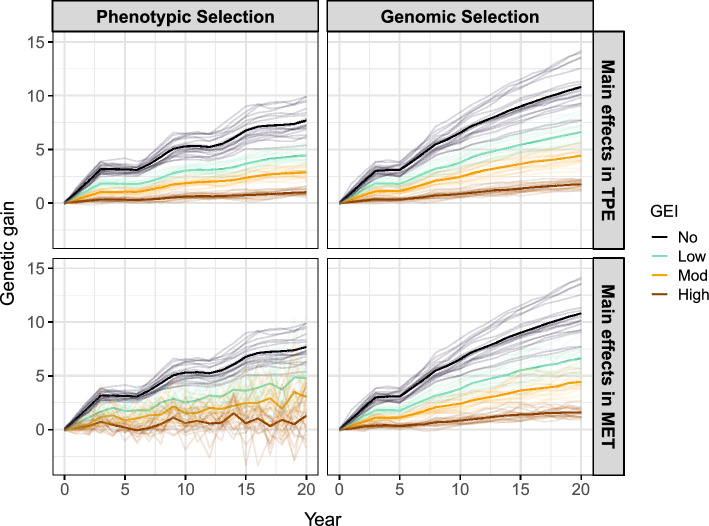
Genetic gain in the simulated plant breeding programmes with phenotypic or genomic selection and no, low, moderate or high GEI. Genetic gain is presented as the mean of the true genotype main effects in the TPE or MET dataset for the headrow stage (*solid lines*), along with individual simulation replicates (*faded lines*). Note: The MET dataset is constructed with one environment for phenotypic selection or 60 environments (3 years) for genomic selection

**Fig. 8:**
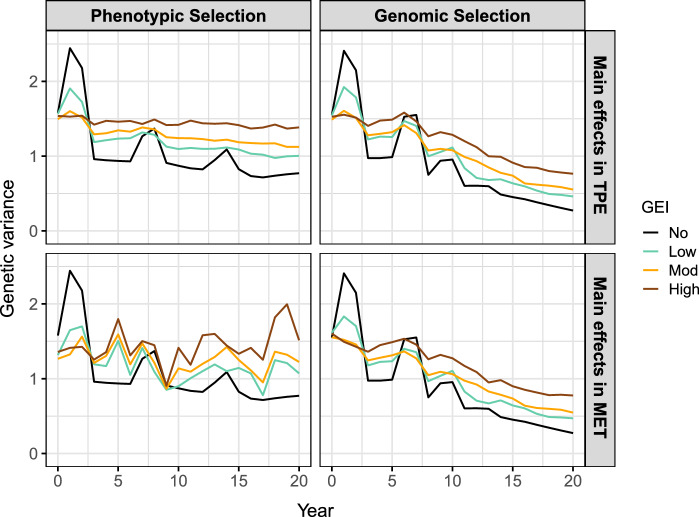
Genetic variance in the simulated plant breeding programmes with phenotypic or genomic selection and no, low, moderate or high GEI. Genetic variance is presented as the variance of the true genotype main effects in the TPE or MET dataset for the headrow stage. Note: The MET dataset is constructed with one environment for phenotypic selection or 60 environments (3 years) for genomic selection

#### Breeding programme comparison

**Fig. 9:**
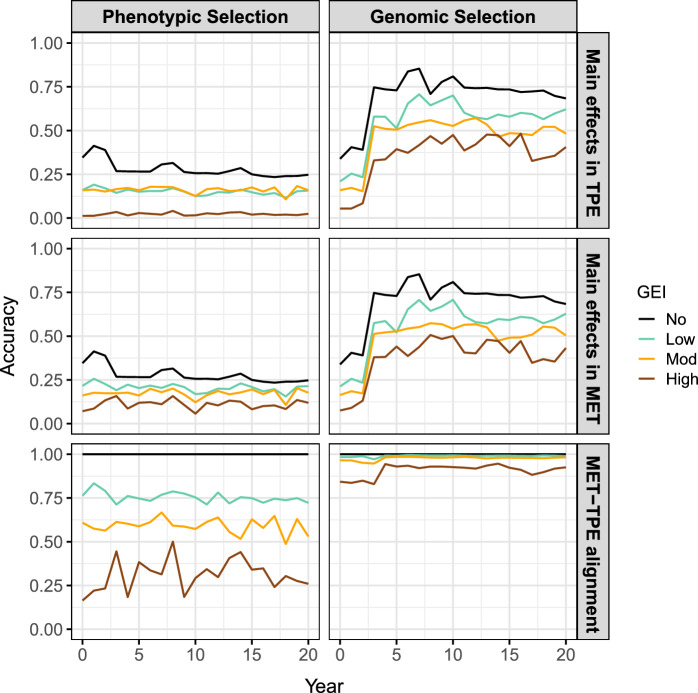
Measures of accuracy in the simulated plant breeding programmes with phenotypic or genomic selection and no, low, moderate or high GEI. The top two panels show the genotype main effect accuracy in the TPE and MET dataset for the headrow stage while the bottom panel shows the correlation between the true main effects in the MET and TPE, referred to as the MET-TPE alignment. Note: The MET dataset is constructed with one environment for phenotypic selection or 60 environments (3 years) for genomic selection

This section compares phenotypic and genomic selection strategies using a breeding programme simulation in AlphaSimR built on the workflow above. The key features of the breeding programme are presented in Fig. 6 and detailed in the Supplementary Material. The breeding programme was simulated with no, low, moderate or high GEI, and then phenotypic or genomic selection was applied for 20 years of breeding. This produced eight scenarios that were replicated 20 times. The MET dataset for phenotypic selection comprised the subset of sampled environments for each stage in the current year only (ranging from one for headrow to 20 for the elite yield trial) and for genomic selection comprised all stages and sampled environments from the past 3 years (60 in total). Phenotypic selection was performed on the phenotype means across environments while genomic selection was performed on the predicted genotype main effects obtained from a compound symmetry model. The aim of the simulation was to track genetic gain, genetic variance and measures of accuracy in the headrow stage during the 20 years of breeding (Figs. 7, 8 and 9). The scenario without GEI was used as a baseline for comparison (*solid black line*).

There are three general results for the genetic gain in Fig. 7:

1. The genetic gain decreases as the level of GEI increases. The genetic gain after 20 years is $\sim$40% lower than the baseline for low GEI, $\sim$60% lower for moderate GEI and more than 80% lower for high GEI.
2. The genetic gain from genomic selection is $\sim$50–70% higher than phenotypic selection in the TPE after 20 years. The largest difference occurs for high GEI.
3. There are negligible differences between the genetic gain in the MET dataset and TPE for genomic selection, but there are noticeable differences for phenotypic selection. The largest difference occurs for high GEI where the genetic gain in the MET is $\sim$30% higher than the TPE after 20 years.

There are three general results for the genetic variance in Fig. 8:

1. The loss in genetic variance decreases as the level of GEI increases. The loss in genetic variance after 20 years is $\sim$30% lower than the baseline for low GEI, $\sim$50% lower for moderate GEI and $\sim$80% lower for high GEI.
2. The loss in genetic variance from genomic selection is 1.0–4.4 times higher than phenotypic selection in the TPE after 20 years. The largest difference occurs for low GEI.
3. There are negligible differences between the loss in genetic variance in the MET dataset and TPE for genomic selection, but there are substantial differences for phenotypic selection. The largest difference occurs for high GEI where the loss in genetic variance is $\sim$90% lower than the TPE after 20 years.

There are three general results for the measures of accuracy in Fig. 9:

1. The main effect accuracies and MET-TPE alignment decrease as the level of GEI increases. The lowest accuracies and alignments occur for high GEI.
2. The main effect accuracies and MET-TPE alignment for genomic selection are much higher than for phenotypic selection. The smallest difference occurs for low GEI while the largest difference occurs for high GEI.
3. The MET-TPE alignment for high GEI is much lower and more variable for phenotypic selection than genomic selection.

The genetic gain, genetic variance and measures of accuracy for all stages of the breeding programme are presented in Supplementary Fig. 5a–c.

## Discussion

Simulations are routinely used in plant breeding for comparing various statistical approaches and breeding strategies in real-time. Many of the current simulations, however, do not adequately capture the complexity of GEI in plant breeding. The framework developed in this paper simulates GEI with desirable structure using multiplicative models. The utility of the framework was demonstrated for two working examples that compared different statistical models and breeding strategies subject to low, moderate and high GEI. The framework has been implemented within the R package FieldSimR (Werner et al. 2024).

The framework for simulating GEI can be summarised by four key steps:

1. *Simulate a reduced rank between-environment genetic variance matrix*, $\mathbf {G_e}$, with heterogeneous genetic variances and correlations. Measures of variance explained were developed for tuning the simulation of $\mathbf {G_e}$ to achieve the desired structure.
2. *Decompose* $\mathbf {G_e}$ to obtain the eigenvectors and eigenvalues for the first *k* terms. This produces a set of terms that capture the structure in $\mathbf {G_e}$.
3. *Set environmental covariates* as the eigenvectors and *simulate genotype slopes* based on the eigenvalues. This produces a set of genotype slopes which can be generated with population structure using a breeding simulation package.
4. *Construct GE effects* by multiplying the environmental covariates with the genotype slopes. This produces a set of GE effects which can be scaled to a large number of environments representing a hypothetical breeder’s TPE.

The framework can be embedded within a linear mixed model for simulating MET datasets and integrated into a simulation package for modelling plant breeding programmes. This can be achieved by sampling the GE effects from the TPE and then constructing phenotypes by adding appropriate non-genetic effects.

The framework simulates heterogeneous genetic variances and correlations in $\mathbf {G_e}$ during Step 1. The genetic variances are simulated from a gamma distribution, which generates positive variances with controllable skew and heterogeneity. The genetic correlations are then simulated following Hardin et al. (2013), which generates a reduced rank correlation matrix by adding structured noise to a base correlation function. In this paper, the approach of Hardin et al. (2013) was extended for simulating a between-environment genetic correlation matrix, $\mathbf {C_e}$, with new functionality to reduce the rank and control the skew. Different underlying structures can be applied to $\mathbf {C_e}$ by altering the base function, e.g. autoregressive processes, variance component models and block structures. The new approach can also be used to reproduce, scale or add noise to correlation distributions estimated from empirical MET datasets, making it well suited to many research objectives.

Measures of variance explained were developed to tune the parameters responsible for simulating different structure in $\mathbf {G_e}$. The measures quantify the proportion of genotype main effect and genotype-by-environment interaction variance, which are mostly controlled by the baseline correlation and skew in $\mathbf {C_e}$. New measures were also developed to quantify the proportion of non-crossover and crossover variance. The measures have analogies to the traditional partitioning of the interaction variance into heterogeneity of genetic variance between environments and lack of genetic correlation (see, for example, Dickerson 1962; Cockerham 1963; Muir et al. 1992; Cooper and DeLacy 1994). The key difference here is that the non-crossover variance quantifies the genetic variation in each environment attributed to perfect positive correlation with the genotype main effects across environments, so it comprises the main effect variance plus any additional variance heterogeneity between environments associated with the main effects. The crossover variance then quantifies the remaining genetic variation arising from a lack of perfect positive correlation between the genotype main effects and GE effects in each environment. The non-crossover and crossover variances are mostly controlled by the baseline correlation and skew in $\mathbf {C_e}$, like the main effect and interaction variances, as well as the shape and scale parameters of the gamma distribution. To the authors’ knowledge, this is the first application of the above measures in simulation.

The framework provides functionality to simulate a hypothetical breeder’s TPE, from which many different MET datasets can be sampled. This is achieved by simulating a set of environments in $\mathbf {G_e}$ during Step 1 which represent the full range of potential environmental conditions in the TPE. Environments are then sampled from the TPE to represent the natural sampling which occurs during field evaluation (Comstock and Moll 1963; Nyquist and Baker 1991; Cooper et al. 1993). Measures of expected accuracy and MET-TPE alignment were presented to inform the sampling process and summarise the simulated MET datasets. The sampling process can be tailored to many routine plant breeding scenarios, including the establishment of a breeder’s so-called “home-site” containing all test genotypes or the addition of trial structure such as different experimental designs and different sources of error at each stage (Smith et al. 2021). Further structure can be applied by altering the base function in $\mathbf {C_e}$ to capture genotype-by-year and genotype-by-location interaction and then tailoring the sampling process accordingly. It is also possible to simulate cyclic changes in the composition of the TPE over time, such as decaying genetic correlations between environments or increasing frequencies of extreme environments representing climate change. The new framework provides an informative and accessible approach to explore many different breeding and environmental scenarios in simulation.

### MET dataset simulation

The framework for simulating GEI can be embedded within a linear mixed model to generate realistic MET datasets. This is achieved by combining the simulated GE effects with appropriate non-genetic effects. A linear mixed model was chosen because it provides a flexible basis to build a wide range of MET datasets. Potential features include additive, dominance and epistatic GE effects (Vitezica et al. 2013), pedigree/genomic information (Meuwissen et al. 2001), correlated plot errors capturing spatial variation (Gilmour et al. 1997) and unbalanced experimental designs, including *p*-rep (Cullis et al. 2006) and sparse testing (Jarquin et al. 2020). This provides a general approach for simulating MET datasets that can be tailored to many research objectives.

The framework was demonstrated for comparing eight statistical models fitted to simulated MET datasets with low, moderate and high GEI. There were three general results:

1. Gains in accuracy can be achieved by sampling more environments in the MET dataset from the TPE, despite losses in accuracy due to increasing GEI. This demonstrates the ability of the framework to devise strategies for optimising the construction of MET datasets.
2. The main effect accuracy in the TPE is always lower than in the MET dataset, as a consequence of the imperfect MET-TPE alignment (Cooper et al. 2023). This demonstrates the utility of the framework to compare different statistical approaches for key metrics of interest.
3. Negligible differences were observed between models for the genotype main effects in the TPE, but not for the GE effects in the MET dataset. This demonstrates the flexibility of the framework to evaluate different statistical models for different selection objectives.

The framework for simulating GEI provides opportunities to explore many new MET dataset simulations. An interesting application of active research is extending the concepts of MET-TPE alignment to the GE effects within environments as a novel way to quantify differences in genotype stability (or specific adaptability) between the MET dataset and the TPE.

### Breeding programme simulation

The framework for simulating GEI can be integrated within a simulation package to compare different breeding strategies over time. This is achieved by defining each multiplicative term as a separate trait, rather than defining each environment as a separate trait (see, for example, Liu et al. 2019). There are two appealing features of this approach. Firstly, the reduced rank model only requires a small number of traits (terms) to be simulated, rather than a much larger number across all years of breeding, e.g. 400 traits would be required for the examples in this paper. Secondly, the environmental covariates capture the desired structure of the hypothetical TPE, so they contain the full range of potential environmental conditions across all years of breeding. This allows different environments to be sampled from the full TPE each year, while supplying projections of genetic gain, genetic variance and measures of accuracy for both the MET dataset and TPE.

The framework’s integration within a simulation package was demonstrated by comparing phenotypic and genomic selection strategies subject to no, low, moderate and high GEI. There were three general results:

1. More realistic projections of genetic gain, genetic variance and accuracy can be obtained in terms of the potential GEI patterns in a breeder’s TPE. This demonstrates the ability of the framework to evaluate different plant breeding strategies in terms of key metrics of interest to breeders.
2. Key metrics are available for every stage of a breeding programme in every year of breeding, for many conceivable GEI scenarios. This demonstrates the potential of the framework to optimise or even reorganise a breeding programme (Gaynor et al. 2017).
3. The simulations can be tailored to field testing involving different trial structure, experimental design and sources of error in each stage. This demonstrates the power of the framework to construct a digital twin of real-world multi-environment field trialling systems.

Overall, the framework for simulating GEI provides opportunities to explore many new research objectives through simulation. An important application is the selection for genotype adaptability within specific environments, which generally requires fitting more complex models. In this paper, genomic selection was based on the predicted genotype main effects obtained from a compound symmetry model, since the MET datasets generated each year were too large to fit more complex models in real-time. This reflects an ongoing challenge in many real-world plant breeding programmes, which ultimately reduces their ability to target specific environments within the TPE (Ceccarelli 1994).

### Concluding remarks

Simulation continues to serve as a valuable tool for plant breeders to optimise their breeding programmes. The integration of a general framework for GEI within simulation represents an important advancement for comparing different statistical approaches and breeding strategies in real-time. The framework is intuitive and well aligned with information routinely collected in plant breeding programmes. The new framework has been implemented within FieldSimR, but is also simple to implement into any simulation package which provides the multi-trait functionality, making it an accessible approach for many breeders and researchers to optimise breeding methodologies and obtain more realistic projections of genetic gain.

### Supplementary Information

Below is the link to the electronic supplementary material.

Supplementary file 1 (pdf 874 KB)

## Figures and Tables

**Fig. 10:**
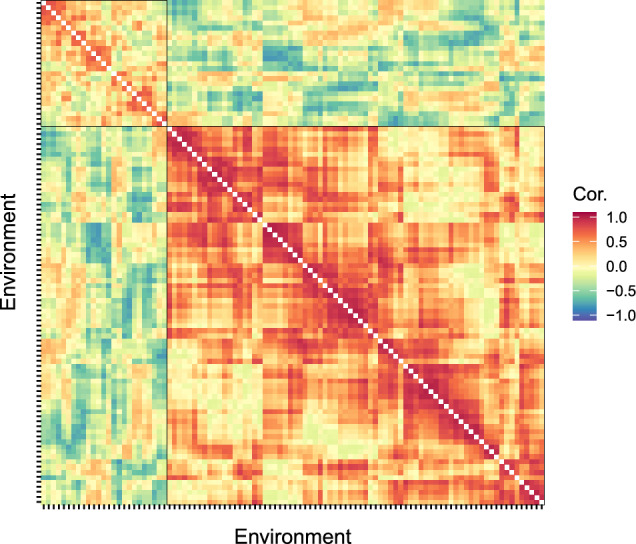
Simulated between-environment genetic correlation matrix with two groups containing 25 and 75 environments, respectively. The *colour key* for the heatmap ranges from 1 (agreement in rankings) through 0 (disagreement in rankings) to $-1$ (reversal of rankings). Note: The groups are completely general and may represent multiple phenotypic traits, multiple TPEs and/or multiple years. The matrix is hierarchically ordered based on separate dendrograms for each group

## Data Availability

The R scripts generated for this study are available at the GitHub repository https://github.com/crWerner/fieldsimr.

## Appendix A: Simulating structured base functions

This appendix extends the between-environment genetic correlation matrix in Eq. (6) to include structured base functions. The between-environment genetic correlation matrix can be generalised as follows:

17 $$\begin{aligned} \mathbf {C_e} = \varvec{\varOmega } + \epsilon \varvec{\varLambda }^{{\!\scriptscriptstyle \top }} \varvec{\varLambda }, \end{aligned}$$

where $\varvec{\varOmega }$ is a $p\times p$ base correlation function, $\epsilon$ controls the magnitude of noise around the base function and $\varvec{\varLambda } = [\varvec{\varLambda }_1 \, \ldots \, \varvec{\varLambda }_p]$ is a matrix of latent covariates in which $\varvec{\varLambda }_{j}$ is the vector for the $j^\textrm{th}$ environment. The form of $\mathbf {C_e}$ in Eq. (6) is obtained by setting $\varvec{\varOmega } = \rho \textbf{J}_p$, where $\rho$ is the baseline genetic correlation and $\textbf{J}_p$ is a $p\times p$ matrix of ones. Other possible base functions include autoregressive processes, variance component models capturing genotype-by-year and genotype-by-location interaction and block structures capturing multiple phenotypic traits, target population of environments (TPE) and/or years. The latter is demonstrated below in terms of multiple generic groups of environments.

### Multiple groups

Assume there are *s* groups with $p_{i}$ environments in the $i^{th}$ group, such that $p = \sum _{i=1}^s p_{i}$ is the total number of environments across all groups. The between-environment genetic correlation matrix can be simulated as follows:

18 $$\begin{aligned} \mathbf {C_e} = \ \rho \textbf{J}_{p} + \oplus ^s_{i=1}\delta _i \textbf{J}_{p_{i}} + \epsilon \varvec{\varLambda }^{{\!\scriptscriptstyle \top }} \varvec{\varLambda }, \end{aligned}$$

where $\varvec{\varOmega } = \rho \textbf{J}_{p} + \oplus ^s_{i=1}\delta _i \textbf{J}_{p_{i}}$ is the base correlation function in which $\delta _i$ is the deviation from the baseline correlation for the $i^{th}$ group and all other parameters are as previously defined. This generates a separate baseline genetic correlation for each group given by $\rho _i=\rho +\delta _i$ and the same baseline genetic correlation between groups given by $\rho$. The reduced rank form of $\mathbf {C_e}$ is achieved by imposing the constraint $0\le \rho _i<1$ where $\rho _i = \rho + \delta$, such that the baseline correlation for each group is equal. The noise is then subject to $\epsilon = 1 - \rho - \delta$ with $\varvec{\Lambda }$ simulated as a $(k-s)\times p$ matrix. If the constraints are not imposed and $(\epsilon < 1 - \rho - \delta )$ or a different baseline correlation is used for each group, i.e. $\rho _i = \rho + \delta _i$, then the diagonal must be constrained to one, and the rank of $\mathbf {C_e}$ will not equal *k*.

The methods above were used to simulated the example of $\mathbf {C_e}$ with two groups presented in Fig. 10. This matrix was constructed with $p_1=25$ environments in group 1 and $p_2=75$ environments in group 2. The baseline genetic correlation was $\rho _1=0.2$ for group 1, $\rho _2=0.4$ for group 2 and $\rho =-0.2$ between groups 1 and 2. The rank of the noise was set to $k=4$, but the overall rank of $\mathbf {C_{e}}$ was 30 because $\rho _1 \ne \rho _2$.

## Appendix B: Measures of variance explained

This appendix derives the variance components for the measures of variance explained in “Measures of variance explained”. The components quantify the magnitude of (i) main effect and interaction variance, (ii) heterogeneity of variance and lack of correlation and (iii) non-crossover and crossover variance simulated in $\mathbf {G_e}$. Without loss of generality, the mean diagonal element of **G** is assumed to be one so that the components derived below represent means across genotypes. All components are derived in terms of the reduced rank model in Eq. (4), which can be considered in terms of the full rank model by setting $k=p$ in the following.

### Main effect and interaction variances

The reduced rank model in Eq. (4) does not have explicit genotype main effects, which instead arise implicitly from the form of $\mathbf {C_e}$ simulated in Eq. (6). Genotype main effects can be obtained after simulation as average across environments, producing genotype-by-environment interaction effects given by the deviations around the main effects in each environment. The main effects and interaction effects are, therefore, given by:

19 $$\begin{aligned} \mathbf {u_g} = \big (\bar{\textbf{s}}_k \otimes \textbf{I}_v\big )\textbf{f}_k \quad \text {and} \quad \mathbf {u_{ge}}&= \textbf{u} - \textbf{1}_p \otimes \mathbf {u_g}, \end{aligned}$$

where $\bar{{\textbf {s}}}_k = {{\textbf {1}}}_p^{\hspace{0.5pt}{\!\scriptscriptstyle \top }}{} {\textbf {S}}_k/p$ is a *k* row-vector of means for each environmental covariate. The interaction effects can be rewritten as $\mathbf {u_{ge}} = \big ({\textbf{S}}_k^* \otimes \textbf{I}_v\big )\textbf{f}_k$, where ${\textbf{S}}_k^* = {\textbf{S}}_k - \bar{\textbf{s}}_k\otimes \textbf{1}_p$ is a $p\times k$ matrix of column centred environmental covariates.

It then follows that:

20 $$\begin{aligned} \left[ \begin{array}{c} \mathbf {u_g} \\ \mathbf {u_{ge}} \end{array}\right] \sim \text{ N }\left( \left[ \begin{array}{c} \textbf{0} \\ \textbf{0} \end{array}\right] , \, \left[ \begin{array}{cc} \bar{\textbf{s}}_k\textbf{L}_k\bar{\textbf{s}}_k^{{\!\scriptscriptstyle \top }} &{}\quad \bar{\textbf{s}}_k\textbf{L}_k{\textbf{S}}_k^{*{\!\scriptscriptstyle \top }} \\ {\textbf{S}}_k^*\textbf{L}_k\bar{\textbf{s}}_k^{{\!\scriptscriptstyle \top }} &{}\quad {\textbf{S}}_k^*\textbf{L}_k{\textbf{S}}_k^{*{\!\scriptscriptstyle \top }} \end{array}\right] \otimes \textbf{G} \right) , \end{aligned}$$

where $\sigma ^2_{g} = \bar{\textbf{s}}_k\textbf{L}_k\bar{\textbf{s}}_k^{{\!\scriptscriptstyle \top }}$ is the genotype main effect variance, which is equal to the mean element of $\mathbf {G_e}$ given by:

21 $$\begin{aligned} \sigma ^2_{g} = \sum _{j=1}^p\sigma _{g_{j}}^2/p^2 + 2\sum ^p_{i < j} \sigma _{g_{ij}}/p^2, \end{aligned}$$

where $\sigma _{g_j}^2$ is the genetic variance of environment *j* and $\sigma _{g_{ij}}$ is the genetic covariance between environments *i* and *j*. The interaction variance is then obtained as the mean diagonal element of ${\textbf{S}}^*_k\textbf{L}_k{\textbf{S}}_k^{*{\!\scriptscriptstyle \top }}$, which is equal to the mean diagonal element of $\mathbf {G_e} - \sigma ^2_g\textbf{J}_p$ given by:

22 $$\begin{aligned} \sigma ^2_{ge} = \sum _{j=1}^p\sigma _{g_{j}}^2/p - \sigma ^2_{g}. \end{aligned}$$

It is important to note that the variance components derived here are built on the known simulation (population) parameters, which differs from the traditional form of these components as a result of sample estimation. In the tradition form, the main effect variance is given by $\sigma ^{*2}_{g}= 2\sum ^p_{i < j} \sigma _{g_{ij}}/p(p-1)$, which is equivalent to the mean *upper triangular* element of $\mathbf {G_e}$. The interaction variance is then given by $\sigma ^{*2}_{ge} = \sum _{j=1}^p\sigma _{g_j}^2/p - \sigma ^{*2}_{g}$, which is equivalent to Eq. 9 in Cooper and DeLacy (1994). FieldSimR provides functionality to obtain both forms of these components.

### Heterogeneity of variance and lack of correlation

The interaction variance can be further partitioned into heterogeneity of genetic variance and lack of genetic correlation (Dickerson 1962; Cockerham 1963; Muir et al. 1992). The heterogeneity of genetic variance is equal to the variance of the standard deviations for each environment (Cooper and DeLacy 1994), which is given by:

23 $$\begin{aligned} \sigma ^2_{ge_h} = \sum _{j=1}^p(\sigma _{g_j} - \bar{\sigma }_g)^2/p, \end{aligned}$$

where $\bar{\sigma }_g=\sum _{j=1}^p\sigma _{g_j}/p$ is the mean standard deviation of the GE effects across environments, which is equal to the mean diagonal element of $\textbf{G}_{\textbf{e}}^{1/2}$.

The lack of genetic correlation arises from a lack of perfect positive correlation between pairs of environments (Cooper and DeLacy 1994), and is given by:

24 $$\begin{aligned} \sigma ^2_{ge_l} = 2\sum ^p_{i < j} \sigma _{g_{i}}\sigma _{g_{j}}(1-\rho _{g_{ij}})/p^2, \end{aligned}$$

where $\rho _{g_{ij}} = \sigma _{g_{ij}}/\sigma _{g_i}\sigma _{g_j}$ is the correlation between the GE effects for environments *i* and *j*, such that $\sigma ^2_{ge_l} = \sigma ^2_{ge} - \sigma ^2_{ge_h}$.

### Non-crossover and crossover variances

The non-crossover variance quantifies the variation in the GE effects for each environment that is attributed to perfect positive correlation with the genotype main effects. The crossover variance then quantifies all remaining variation in the GE effects which arises from a lack of perfect positive correlation with the main effects (Fig. 1, also see Tolhurst 2024).

It is assumed that:

25 $$\begin{aligned} \left[ \begin{array}{c} \mathbf {u_g} \\ \textbf{u} \end{array}\right] \sim \text{ N }\left( \left[ \begin{array}{c} \textbf{0} \\ \textbf{0} \end{array}\right] , \, \left[ \begin{array}{cc} \bar{\textbf{s}}_k\textbf{L}_k\bar{\textbf{s}}_k^{{\!\scriptscriptstyle \top }} &{}\quad \bar{\textbf{s}}_k\textbf{L}_k{\textbf{S}}_k^{{\!\scriptscriptstyle \top }} \\ {\textbf{S}}_k\textbf{L}_k\bar{\textbf{s}}_k^{{\!\scriptscriptstyle \top }} &{}\quad {\textbf{S}}_k\textbf{L}_k{\textbf{S}}_k^{{\!\scriptscriptstyle \top }} \end{array}\right] \otimes \textbf{G} \right) , \end{aligned}$$

where ${\varvec{\Sigma }}^*_{g}= {\textbf{S}}_k\textbf{L}_k\bar{\textbf{s}}_k^{{\!\scriptscriptstyle \top }}$ is a *p*-vector containing the covariances between the genotype main effects and GE effects for each environment, denoted as $\sigma ^*_{g_j}$.

The non-crossover variance is given by:

26 $$\begin{aligned} \sigma ^2_{n} = \sum _{j=1}^p \sigma ^{*2}_{g_j} /p\sigma ^2_{g}, \end{aligned}$$

which is equal to the variation in the GE effects for each environment that is explained by the genotype main effects, i.e. $\sigma ^2_{n} = \sum _{j=1}^p \rho ^{*2}_{g_j}\sigma ^2_{g_j}/p$, where $\rho ^*_{g_j}$ is the correlation between the main effects and GE effects for the $j^{th}$ environment. It follows that $\sigma ^2_{n} \ge \sigma ^2_{g}$, since the non-crossover variance also captures variation in the interaction effects that are explained by the main effects.

The crossover variance is then obtained as:

27 $$\begin{aligned} \sigma ^2_{c} = \sum _{j=1}^p\sigma _{g_j}^2/p - {\sigma }_n^2, \end{aligned}$$

which is equal to all remaining variation in the GE effects that is not explained by the genotype main effects, i.e. $\sigma ^2_{c} = \sum _{j=1}^p(1-\rho ^{*2}_{g_j})\sigma ^2_{g_j}/p$. Note that constraints may be required to ensure the crossover variance also captures any variation in the GE effects which arises from perfect *negative* correlation with the main effects (see Tolhurst 2024, for further details).

## Appendix C: R code to simulate an example TPE and sample a MET dataset

The following base R code simulates a small example TPE with $p=100$ environments, from which a MET dataset is sampled with $p_m=10$ environments. A randomised complete block design is used for each environment with $r=2$ replicate blocks of $v=400$ genotypes.

The R code is also provided in Supplementary Script S0.
