# Supplementary material for: A framework for simulating genotype-by-environment interaction using multiplicative models
Source: Theor Appl Genet. 2024 Aug 6;137(8):197. doi: 10.1007/s00122-024-04644-7 (PMC11303478; doi:10.1007/s00122-024-04644-7)

Supplementary Material

A framework for simulating genotype by environment interaction using multiplicative models

J. Bančič, G. Gorjanc, D.J. Tolhurst

https://doi.org/10.1007/s00122-024-04644-7

Description of a line breeding programme simulation

A 20-year line breeding programme is simulated with phenotypic and genomic selection strategies using AlphaSimR (Gaynor et al., 2017). The simulation begins by generating 30 founder parents through the default wheat species history in AlphaSimR (see Gaynor et al., 2017, for full details). The hypothetical trait is assumed to consist of 300 QTLs and 600 SNPs per chromosome. A breeding cycle entails the crossing of 30 parents to create 100 full-sib families with 100 doubled haploid (DH) lines per family (10,000 DH lines in total). The progression towards a new release variety involves four successive stages: head-to-row (HDRW), preliminary yield trial (PYT), advanced yield trial (AYT), and elite yield trial (EYT). These stages contain different numbers of genotypes, environments, replicates per environment and error variances to mimic a plausible plant breeding programme. The key components of the line breeding programme are presented in Fig. 6 of the manuscript.

For each year of the phenotypic selection strategy, the 10 DH lines reaching the EYT stage replace the oldest 10 parents in the crossing block. Conversely, the 10 new parents for the genomic selection strategy are selected from the HDRW stage. Another important difference is that the genomic selection strategy employs a compound symmetry model to obtain predicted genotype main effects for selection at each stage, including (i) selection of parents from the HDRW stage (ii) advancement of DH lines from the HDRW to the PYT stage without phenotypic data and (iii) genomic-assisted selection in the PYT and AYT stages. The training population is initially collected for 3 years prior to commencing genomic selection, and then maintained using a rolling window of 3 years of phenotypic and genomic data. The results and figures in the manuscript for both selection strategies correspond to the 20 years of breeding after the initial 3-year period.

The simulation of phenotypic and genomic selection strategies were tested under no, low, moderate and high GEI, with each strategy replicated 20 times. The scenario labelled as 'no GEI' resembles a typical plant breeding simulation that does not consider GEI, while the scenarios labelled as ‘low, moderate or high GEI’ implement the new framework developed in the manuscript. The between-environment genetic variance matrices, $\mathbf{G_e}$, are summarised in Tbl. 1 and presented in Figs. 2 and 3. Note that the GEI patterns in $\mathbf{G_e}$ represent the full set of expected patterns in the TPE with reference to the founder population, that is prior to crossing and selection. The observed GEI patterns at any point in time are then dictated by (i) the sampled environments in the current year and (ii) the structure of the breeding population.

Genetic gain, genetic variance and measures of accuracy are tracked over time.

1. **Genetic gain** is taken as the average of the true genotype main effects in the TPE or MET dataset. The genetic gain in the TPE at any point in time is given by $\mu_g^* = \bar{\mathbf{s}}_k \boldsymbol{\tau}_{\mathbf{s}_k}^*$, where $\bar{\mathbf{s}}_k$ is the k row-vector of means for each environmental covariate and $\boldsymbol{\tau}_{\mathbf{s}_k}^*$ is the k-vector of means for the current genotype slopes, $\mathbf{f}_k^*$. Initially, $\mathrm{E}(\mathbf{f}_k) = \mathbf{0}$ in the founder population so that $\boldsymbol{\tau}_{\mathbf{s}_k} = \mathbf{0}$, but this changes over time due to selection and crossing. A similar measure is obtained for the MET dataset by considering those environments sampled in each MET.

2. **Genetic variance** is taken as the variance of the true genotype main effects in the TPE or MET dataset. The genetic variance in the TPE at any point in time is given by $\sigma_g^{*2} = \bar{\mathbf{s}}_k \mathbf{L}_k^* \bar{\mathbf{s}}_k^{\top}$, where $\mathbf{L}_k^*$ is the $k \times k$ matrix containing the variances and covariances of the current genotype slopes. Initially, $\mathrm{var}(\mathbf{f}_k) = \mathbf{L}_k$ in the founder population, where $\mathbf{L}_k$ is a diagonal matrix, but the variances change over time and covariances are induced between terms due to selection and crossing. A similar measure is obtained for the MET dataset by considering those environments sampled in each MET.

3. **Measures of accuracy** include the main effect accuracies in the TPE and MET dataset as well as the MET-TPE alignment.
   a. The main effect accuracy in the TPE at any point in time is given by the empirical correlation between the true and predicted genotype main effects, which can be written as $\mathrm{cor}(\mathbf{u}_{\mathbf{g}}^*, \tilde{\mathbf{u}}_{\mathbf{g}_m}^*)$, where $\mathbf{u}_{\mathbf{g}}^*$ is the v-vector of current main effects in the TPE and $\tilde{\mathbf{u}}_{\mathbf{g}_m}^*$ is the v-vector of main effects predicted from the MET dataset. A similar measure is obtained for the MET dataset using the true genotype main effects across those environments sampled in each MET, denoted by $\mathbf{u}_{\mathbf{g}_m}^*$.
   b. The MET-TPE alignment at any point in time is given by the empirical correlation between the true genotype main effects in the MET and TPE, which can be written as $\mathrm{cor}(\mathbf{u}_{\mathbf{g}_m}^*, \mathbf{u}_{\mathbf{g}}^*)$.

Sup. Fig. 1: Parameters responsible for the structure of the between-environment genetic correlation matrix, $\mathbf{C_e}$. The parameters $(\rho, \epsilon, \gamma, k)$ were altered, while keeping the others constant ($\rho = 0,\ \ \epsilon = 1 - \rho,\ \gamma = 0,\ k = 7$). Different between-environment genetic variance matrices, $\mathbf{G_e}$, were then obtained by multiplying each $\mathbf{C_e}$ with $\mathbf{D_e}$ in Fig. 2 of the manuscript using Eq. 5, from which measures of variance explained were calculated as labelled above each histogram. The *vertical blue line* represents the mean genetic correlation between environments (also labelled).

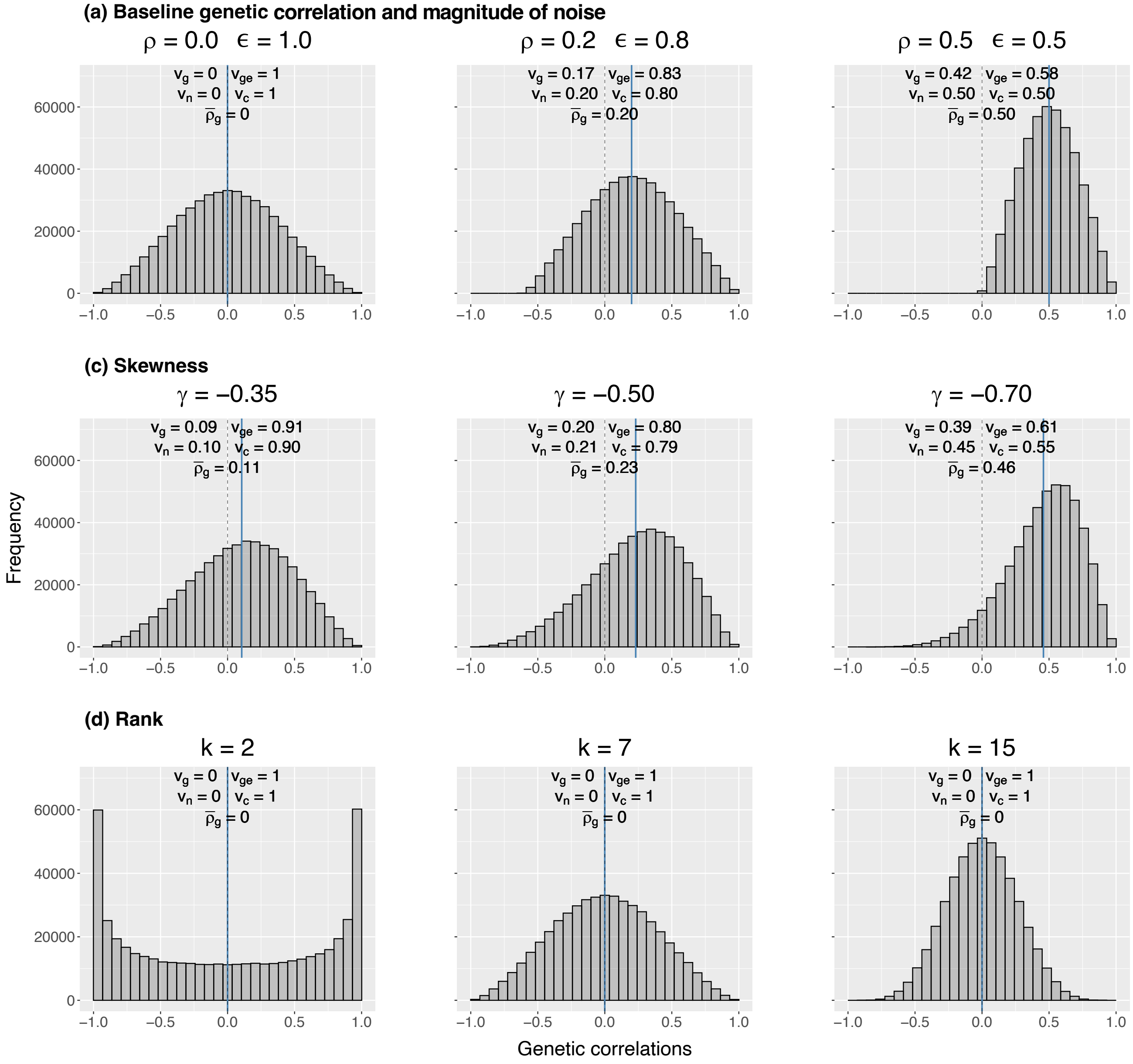

Sup. Fig. 2: Expected main effect accuracy in the TPE and MET, and the expected MET-TPE alignment for different proportions of genotype main effect variance (v_g) and different numbers of environments sampled in the MET dataset (p_m). Highlighted are the expected trajectories for $p_m = 5,\ 10,\ 20$ and 50 environments. The overall plot-level heritability is $\mathrm{H}^2 = 0.3$ (i.e. $\bar{\sigma}_g^2 = 1.47$ and $\sigma_\varepsilon^2 = 3.44$), with two replicates per environment ($r = 2$).

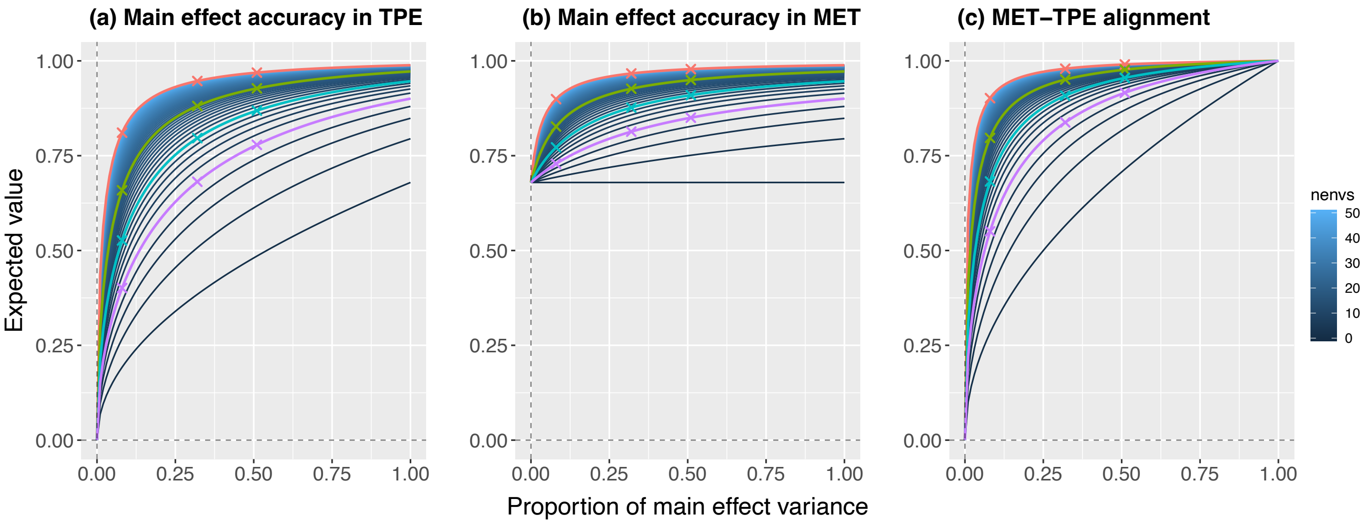

Note: The *crosses* represent the expected values for the three examples of low, moderate and high GEI used in the manuscript, i.e. for proportions of main effect variance of $v_g = 0.51,\ 0.32$ and 0.08, respectively (see Tbl. 1. and Fig. 5). A 'no GEI' scenario would be equivalent to $v_g = 1$.

The proportion of main effect variance is given by Eq. 7 while the expected main effect accuracy in the TPE is given by Eq. 12, which is equal to the expected main effect accuracy in the MET (Eq. 13) multiplied by the expected MET-TPE alignment (Eq. 14). The measures of accuracy are presented in the following figures for 1000 simulated MET datasets with low, moderate and high GEI. Note that the MET-TPE alignments in these figures are built using Eq. 13, and are therefore different to those presented in Fig. 5 of the manuscript which are built using the empirical correlation between the true genotype main effects in the TPE and those sampled in each MET dataset.

Sup. Fig. 3a: True simulation parameters for 1000 MET datasets with 5, 10, 20 or 50 environments sampled from a TPE with low GEI. The top panel presents the proportion of main effect (v_g) and interaction (v_{ge}) variances, proportion of non-crossover (v_n) and crossover (v_c) variances and the plot-level heritability (H^2). The middle panel presents the mean genetic $(\bar{\sigma}_g^2)$ and error (σ_ε^2) variances. The bottom panel presents the main effect accuracies in the TPE (r_g) and MET (r_m), the MET-TPE alignment (r_{mt}) and the accuracy of the GE effects in the MET (r_{ge}) per Eqs. 12-16 of the manuscript. Note: The *crosses* represent the expected values per Tbl. 2.

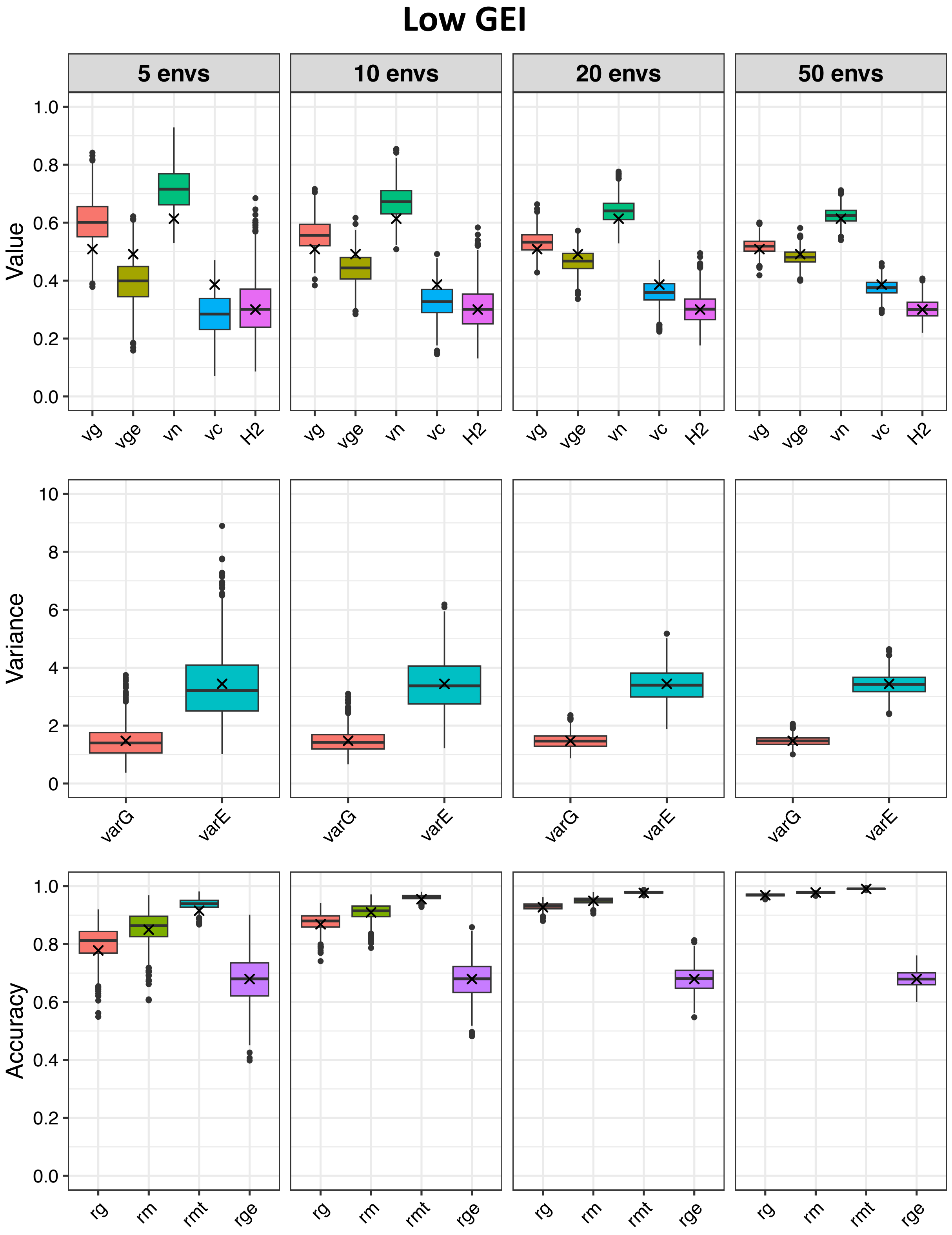

Sup. Fig. 3b: True simulation parameters for 1000 MET datasets with 5, 10, 20 or 50 environments sampled from a TPE with moderate GEI. The top panel presents the proportion of main effect (v_g) and interaction (v_{ge}) variances, proportion of non-crossover (v_n) and crossover (v_c) variances and the plot-level heritability (H^2). The middle panel presents the mean genetic $(\bar{\sigma}_g^2)$ and error (σ_ε^2) variances. The bottom panel presents the main effect accuracies in the TPE (r_g) and MET (r_m), the MET-TPE alignment (r_{mt}) and the accuracy of the GE effects in the MET (r_{ge}) per Eqs. 12-16 of the manuscript. Note: The *crosses* represent the expected values per Tbl. 2.

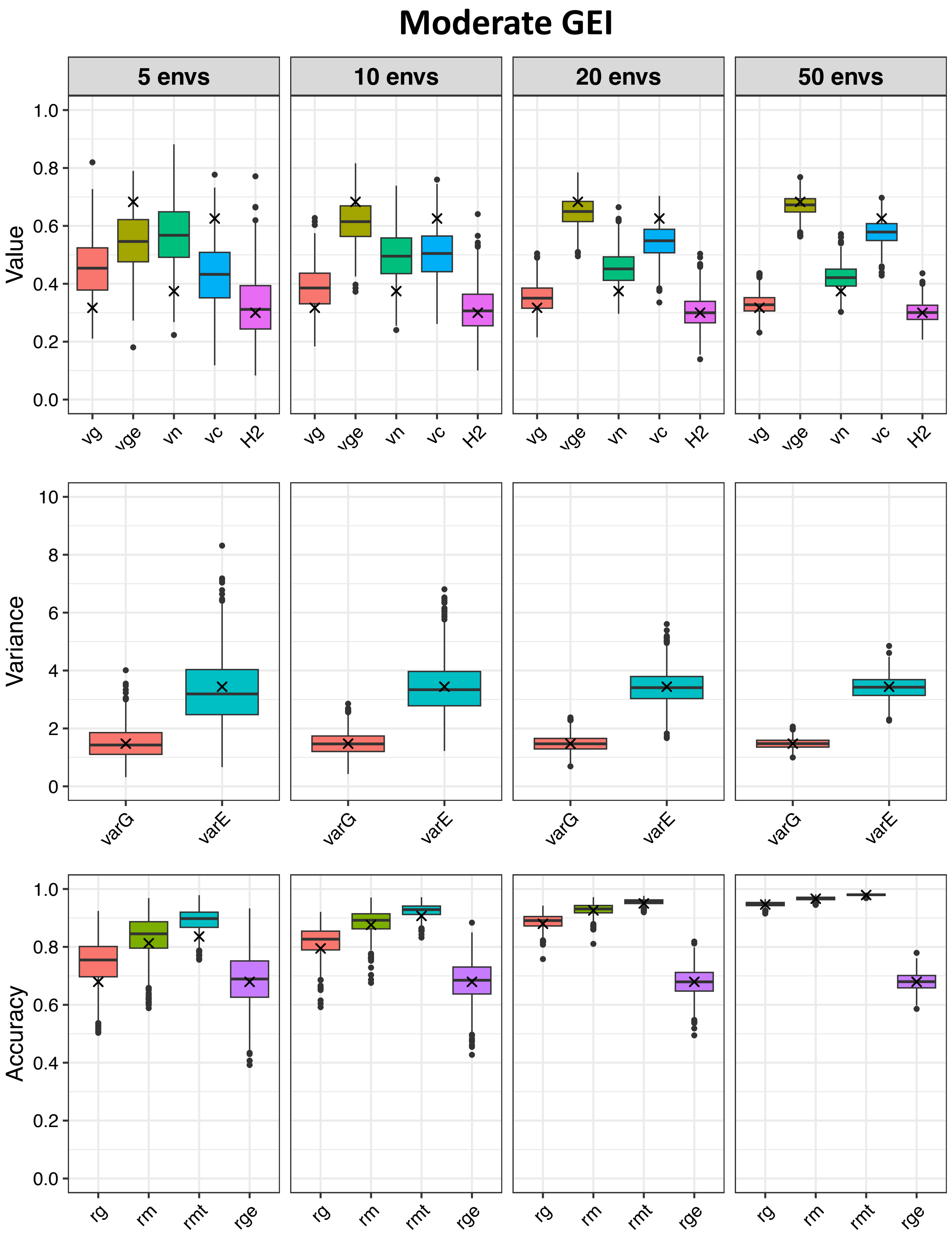

Sup. Fig. 3c: True simulation parameters for 1000 MET datasets with 5, 10, 20 or 50 environments sampled from a TPE with high GEI. The top panel presents the proportion of main effect (v_g) and interaction (v_{ge}) variances, proportion of non-crossover (v_n) and crossover (v_c) variances and the plot-level heritability (H^2). The middle panel presents the mean genetic $(\bar{\sigma}_g^2)$ and error (σ_ε^2) variances. The bottom panel presents the main effect accuracies in the TPE (r_g) and MET (r_m), the MET-TPE alignment (r_{mt}) and the accuracy of the GE effects in the MET (r_{ge}) per Eqs. 12-16 of the manuscript. Note: The *crosses* represent the expected values per Tbl. 2.

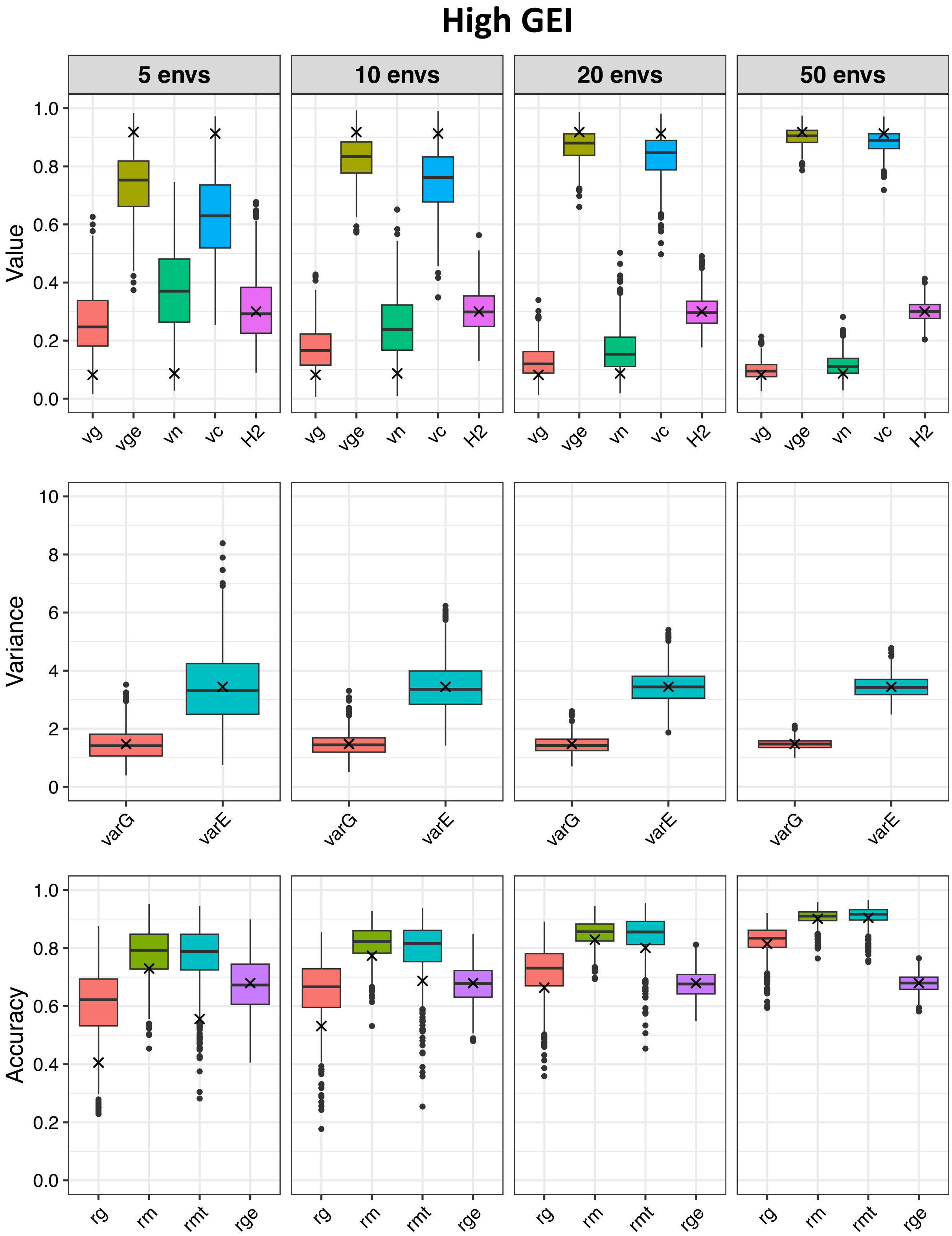

Description of statistical models fitted to simulated MET datasets

Eight linear mixed models were fitted to the 1000 simulated MET datasets with low, moderate and high GEI. It is important to note that the primary objective here is to demonstrate the potential application of the new framework, rather than recommend specific model formulations.

All linear mixed models fitted fixed environments and random genotype effects. Strictly, the environment factor should also be treated as random to assess uncertainty when projecting the results to the TPE. However, a factor analytic model with random genotypes and environments cannot be fitted in a frequentist setting, which is required here. Furthermore, fitting random environments and fixed genotypes was not feasible, particularly for the 5 and 10 environment scenarios.

The eight linear mixed models fitted different variance structures for the GE effects using ASReml-R (Butler et al., 2023):

1. **Main effects (Main):** included genotype main effects modelled by a single variance component.
   a. The genotype main effects were obtained directly from the fit of ASReml-R.
   b. The GE effects were obtained by repeating the main effects across all environments.

2. **Compound symmetry (Comp):** included genotype main effects and genotype by environment interaction effects, each modelled by a single variance component.
   a. The genotype main effects were obtained directly from the fit of ASReml-R.
   b. The total GE effects were obtained by summing the main effects and interaction effects obtained from ASReml-R.

3. **Main effects plus diagonal (MDiag):** included genotype main effects and genotype by environment interaction effects, now modelled by a separate variance component for each environment.
   a. The genotype main effects were obtained directly from the fit of ASReml-R.
   b. The total GE effects were obtained by summing the main effects and interaction effects obtained from ASReml-R.

4. **Diagonal (Diag):** included GE effects modelled by a separate variance component for each environment.
   a. The genotype main effects were obtained by averaging across the GE effects for each environment.
   b. The GE effects were obtained directly from the fit of ASReml-R.

5-8. **Factor analytic (FA):** included common GE effects modelled by a reduced rank (RR) model and specific GE effects modelled by a diagonal (DIAG) model (not fitted for order 3 and 4 for the 5 environment scenario).
   a. The genotype main effects were obtained by averaging across the common GE effects for each environment.
   b. The common GE effects were obtained directly from the fit of ASReml-R.

   The specific GE effects can be considered for a. and b. where appropriate.

Sup. Fig. 4a: Reliabilities of parameter estimates for seven statistical models fitted to 1000 simulated MET datasets with low, moderate or high GEI and 5, 10, 20 or 50 environments. Presented is the average root mean square error of the estimated genetic variances, covariances and correlations with 80% sample quantiles.

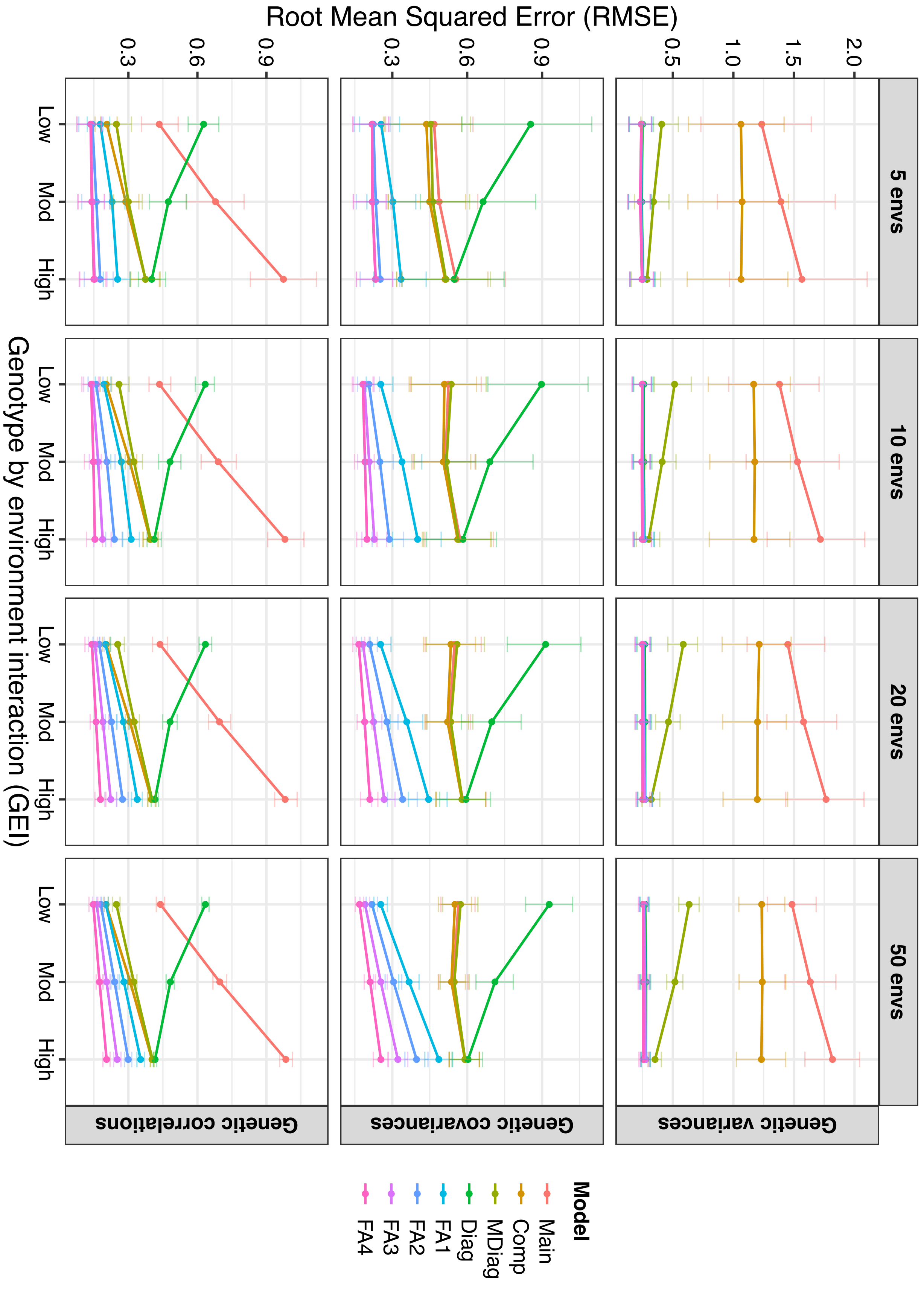

Sup. Fig. 4b: Model fit criteria for eight statistical models fitted to 1000 simulated MET datasets with low, moderate or high GEI and 5, 10, 20 or 50 environments. Presented is the average AIC, proportion of variance explained and model run times in ASReml-R (Butler et al., 2023) with 80% sample quantiles.

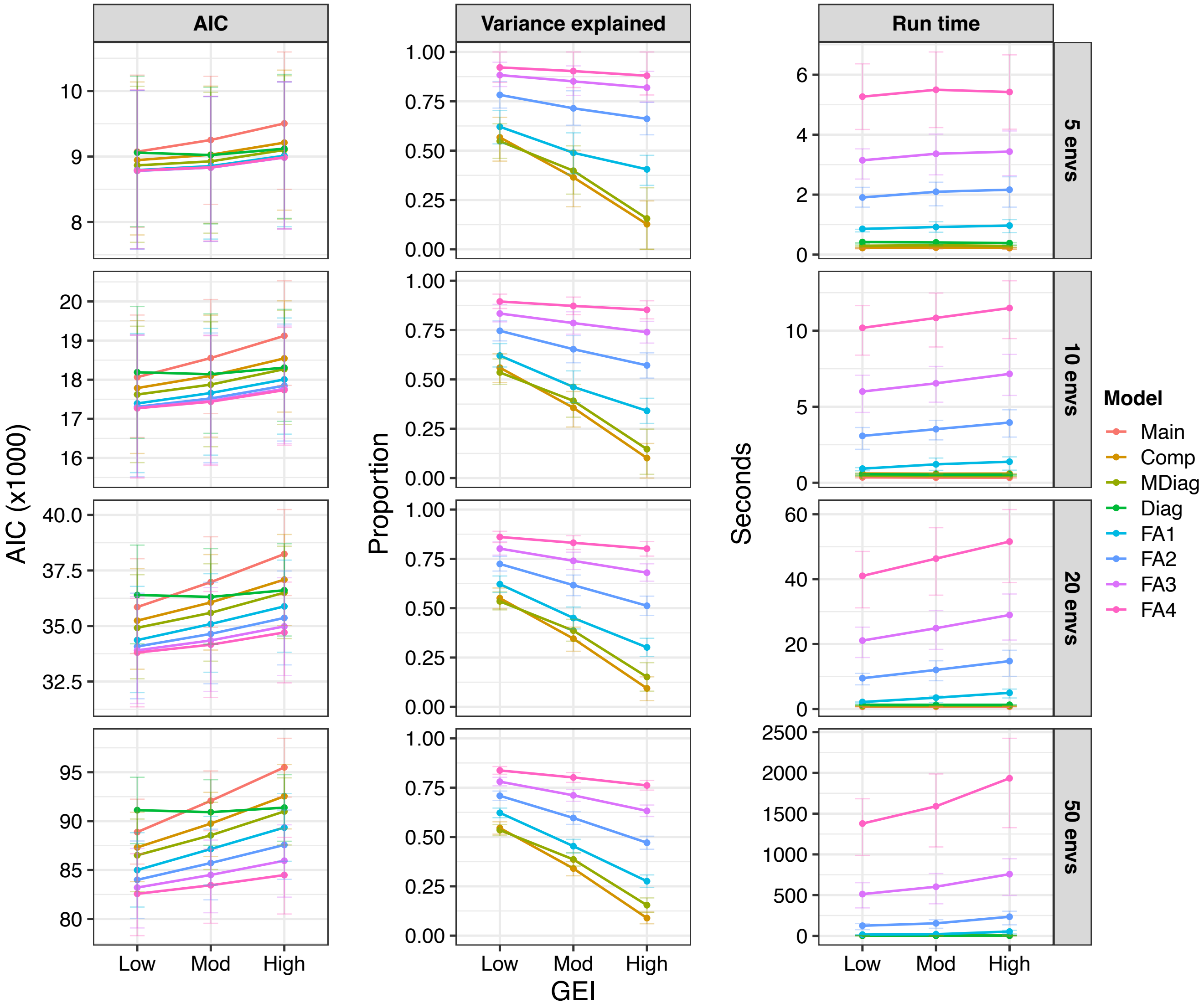

Note: The proportion of variance explained is calculated as the proportion of genotype main effect variance of the overall genetic variance for the compound symmetry and main effects plus diagonal models, or the proportion of variance explained by the common factors for the factor analytic models. This measure is not available for the main effects and diagonal models.

The factor analytic models were fitted sequentially, with the FAk model used to obtain start values for the FA$(k+1)$ model. The run times are therefore presented as cumulative times, such that the FA4 model includes the run times of the FA3, FA2 and FA1 models.

Sup. Fig. 5a: Genetic gain, genetic variance and accuracy in the simulated line breeding programme with low GEI for all stages. Note: 10 new parents are selected each year from the EYT stage for phenotypic selection or the HDRW stage for genomic selection.

Low GEI

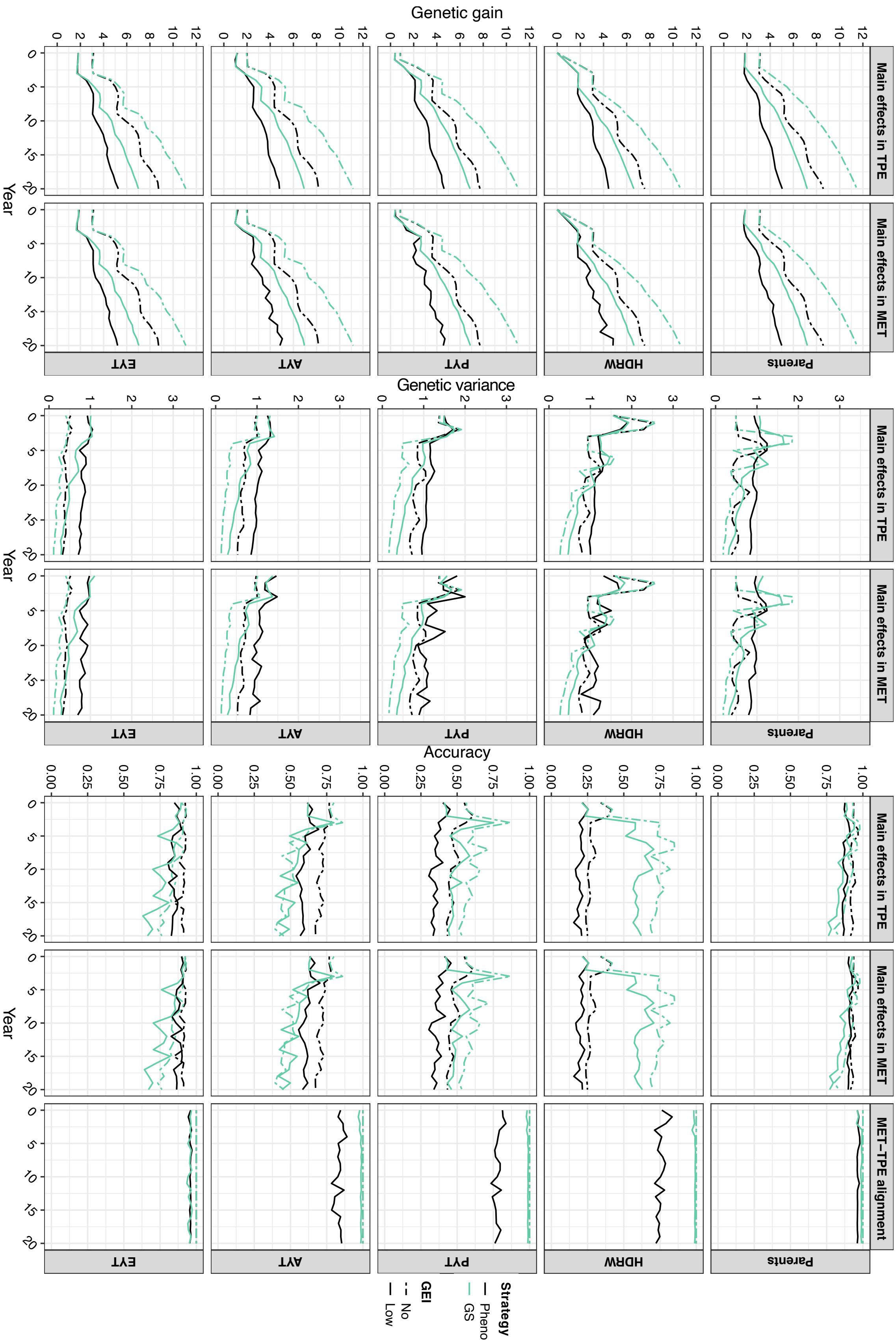

Sup. Fig. 5b: Genetic gain, genetic variance and accuracy in the simulated line breeding programme with moderate GEI for all stages. Note: 10 new parents are selected each year from the EYT stage for phenotypic selection or the HDRW stage for genomic selection.

Moderate GEI

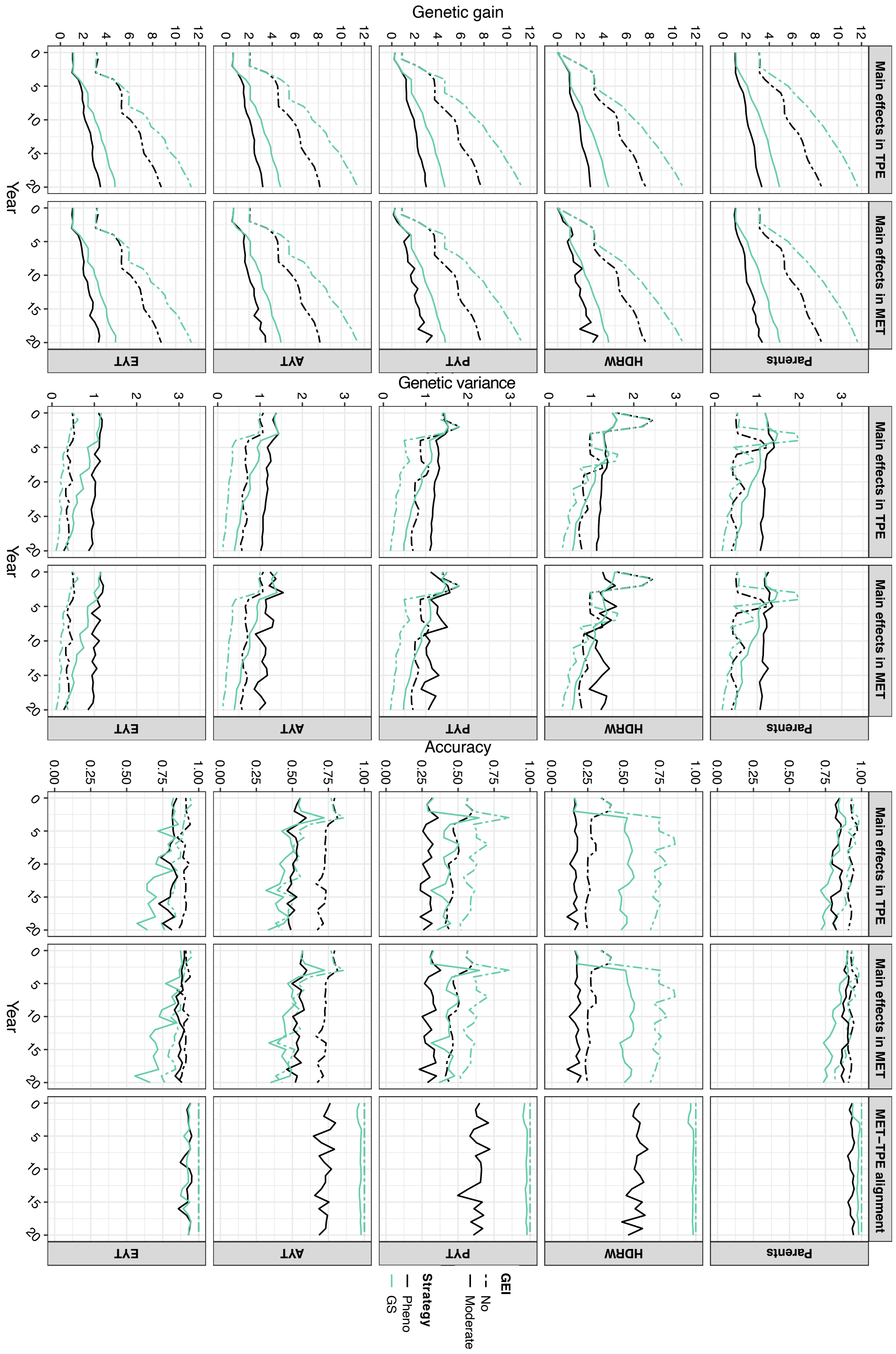

Sup. Fig. 5c: Genetic gain, genetic variance and accuracy in the simulated line breeding programme with high GEI for all stages. Note: 10 new parents are selected each year from the EYT stage for phenotypic selection or the HDRW stage for genomic selection.

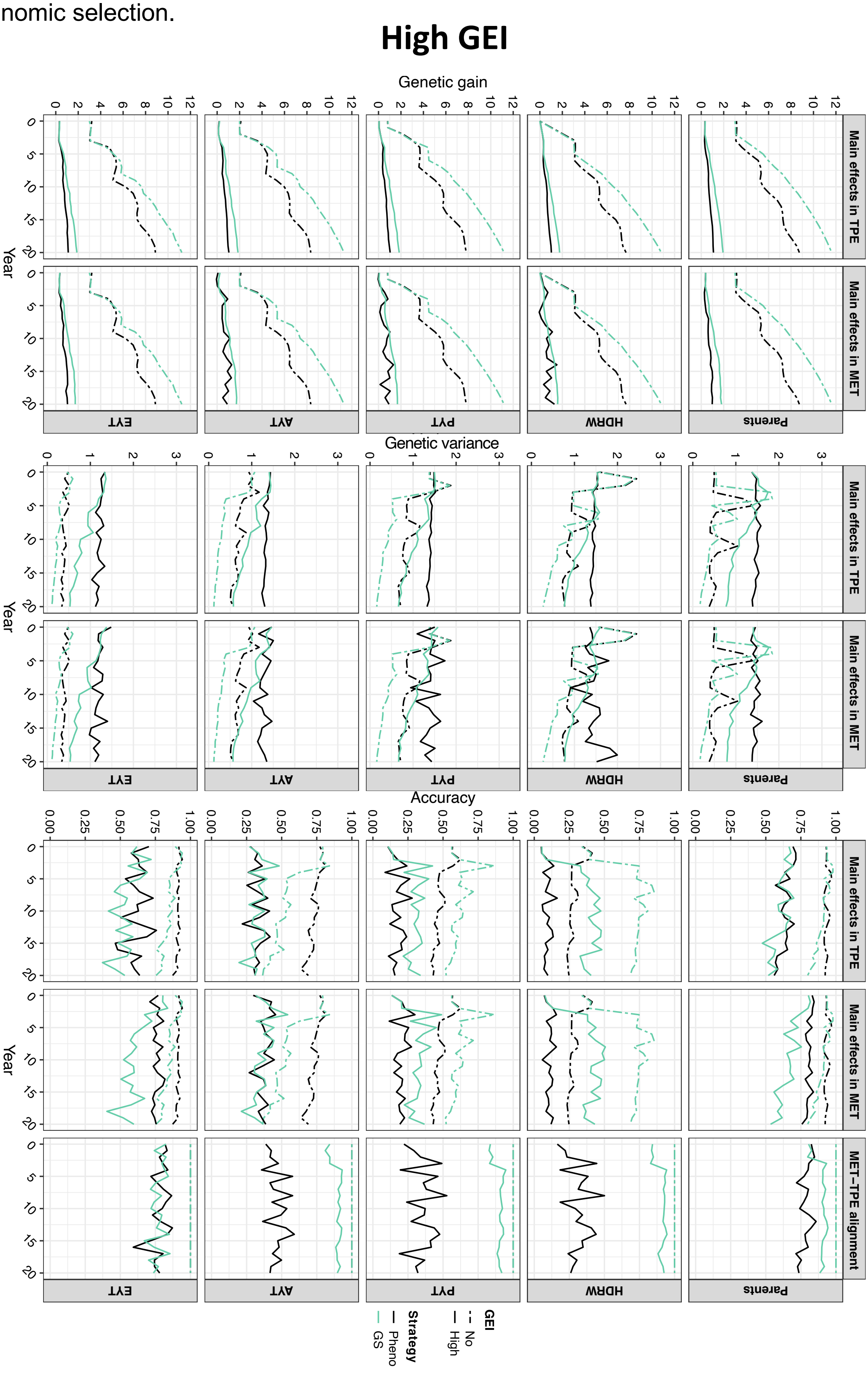

Supplement: Supplementary file 1 — (pdf 874 KB) [file 122_2024_4644_MOESM1_ESM.pdf]
